# KLF3 promotes the 8‐cell‐like transcriptional state in pluripotent stem cells

**DOI:** 10.1111/cpr.12914

**Published:** 2020-09-29

**Authors:** Jing Hao, Xi Yang, Chao Zhang, Xue‐Tao Zhang, Ming Shi, Shao‐Hua Wang, Li Mi, Yu‐Ting Zhao, Huiqing Cao, Yangming Wang

**Affiliations:** ^1^ Beijing Key Laboratory of Cardiometabolic Molecular Medicine Institute of Molecular Medicine Peking University Beijing China; ^2^ Academy for Advanced Interdisciplinary Studies Peking University Beijing China

**Keywords:** early embryo development, embryonic stem cells, heterogeneity, Klf3, protein degradation

## Abstract

**Objectives:**

Mouse embryonic stem cell (mESC) culture contains various heterogeneous populations, which serve as excellent models to study gene regulation in early embryo development. The heterogeneity is typically defined by transcriptional activities, for example, the expression of Nanog or Rex1 mRNA. Our objectives were to identify mESC heterogeneity that are caused by mechanisms other than transcriptional control.

**Materials and methods:**

Klf3 mRNA and protein were analysed by RT‐qPCR, Western blotting or immunofluorescence in mESCs, C2C12 cells, early mouse embryos and various mouse tissues. An ESC reporter line expressing KLF3‐GFP fusion protein was made to study heterogeneity of KLF3 protein expression in ESCs. GFP‐positive mESCs were sorted for further analysis including RT‐qPCR and RNA‐seq.

**Results:**

In the majority of mESCs, KLF3 protein is actively degraded due to its proline‐rich sequence and highly disordered structure. Interestingly, KLF3 protein is stabilized in a small subset of mESCs. Transcriptome analysis indicates that KLF3‐positive mESCs upregulate genes that are initially activated in 8‐cell embryos. Consistently, KLF3 protein but not mRNA is dramatically increased in 8‐cell embryos. Forced expression of KLF3 protein in mESCs promotes the expression of 8‐cell‐embryo activated genes.

**Conclusions:**

Our study identifies previously unrecognized heterogeneity due to KLF3 protein expression in mESCs.

## Graphical abstract

Klf3 mRNA is expressed ubiquitously during early mouse embryo development. However, KLF3 protein is only expressed in 8‐cell and morula stages. Interestingly, a subset of embryonic stem cells express high level of KLF3 protein, which upregulates numerous 8‐cell‐embryo activated genes.

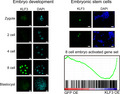

## 1 INTRODUCTION

Embryonic stem cells (ESCs) are derived from the inner cell mass of a blastocyst embryo and can self‐renew indefinitely while retaining ability to differentiate into all other cells in the body.^1^ For these remarking properties, they have played pivot roles in the development of regenerative medicine and served as great model systems to understand early embryonic development. Traditionally, ESCs are used to study tissue formation and specialization of functional cells such as neurons and cardiomyocytes. Interestingly, multiple distinct subpopulations were recently discovered in mESC cultures,^2^, ^3^, ^4^, ^5^ demonstrating the heterogeneity of mESCs. In particular, ~0.5% cells in serum cultured mESCs are found to activate 2‐cell embryo specific genes including retrotransposons MERVL and Zscan4.^6^, ^7^ Since then, the transition between ordinary mESCs and these special 2‐cell‐like cells have been adopted as a model system to study early events in 2‐cell embryos.^8^, ^9^, ^10^, ^11^, ^12^ These studies have provided significant insights on the transcriptional regulation during zygotic genome activation. However, an important question remains whether cell populations resembling other early embryonic stages prior to blastocyst development exist.

Embryonic stem cell fate is determined by multiple layers of regulation including epigenetics, transcription and translation. Accordingly, previous studies have identified a number of key regulators including chromatin modifiers, transcription factors (TFs), RNA binding proteins and non‐coding RNAs in ESCs. Sitting at the core of ESC transcriptional network are TFs Oct4 (also known as Pou5f1), Sox2 and Nanog.^13^ In addition, protein interactome studies followed by functional dissection have revealed that many other TFs work together with these core TFs to modulate ESC fate.^14^, ^15^, ^16^ Interestingly, the activity of promoters or enhancers of some TFs such as Nanog^2^ and Rex1 (also known as Zfp42)^4^ displays various degrees of heterogeneity in mESC cultures. While the relevance of heterogeneity in vitro to the cell fate determination in vivo is still in debate, studying the phenomenon has provided plenty insights on gene regulation in mESCs. Moreover, the inconsistency between RNA and protein expression is observed in around 50% differentially regulated genes during mESC differentiation,^17^ suggesting high prevalence of translation and post‐translational regulation in mESCs. Recent studies at systemic level or on individual genes find that post‐translational regulation,^18^, ^19^, ^20^, ^21^ in particular protein degradation, may play important roles in mESC fate determination. Nevertheless, mESC heterogeneity due to protein stability control has not been reported.

Kruppel‐like factor (KLF) family transcription factors play important roles in diverse physiologic and pathologic conditions.^22^, ^23^ The family has 17 members sharing highly conserved DNA‐binding domain, which locates at the C terminal and consist of three C2H2 zinc fingers. In contrast, the N‐terminal domain of KLF proteins is more variable, which recruit different coregulators to exert diverse biological functions. Klf2, Klf4 and Klf5 have been shown as essential transcription factors for pluripotency maintenance in mESCs.^21^, ^24^, ^25^ Among them, Klf4 has been used as one of the four reprogramming factors in the first study of induced pluripotent stem cells.^26^ However, except for Klf2, Klf4 and Klf5, the function for most other KLF proteins is still unknown in mESCs.

In the present study, we find that Klf3 mRNA is expressed but its protein is repressed in mESCs. KLF3 protein is translated but vigorously degraded due to its highly proline‐rich sequence, which leads to an internally disordered structure. Surprisingly, we find that KLF3 protein is expressed in a very small subset of mESCs, which upregulates numerous genes that are initially activated during 4‐ to 8‐cell embryo stages. Interestingly, KLF3 protein but not mRNA is also highly upregulated in 8‐cell embryos. Importantly, KLF3 protein may play a functional role in activating these transcripts, supported by the evidence that forced expression of KLF3 protein upregulates 8‐cell‐embryo activated transcripts. Together, this study highlights the importance of post‐translational regulation in early embryo development and identifies a distinctive population of mESCs marked by KLF3 protein expression.

## 2 MATERIALS AND METHODS

### 2.1 Cell culture

mESCs were grown in high glucose DMEM (Hyclone, Cat. #30045.10) containing 0.1 mmol/L non‐essential amino acids (Gibco, Cat. #11140050), 1 mmol/L L‐glutamine (Gibco, Cat. #25030081), 15% foetal bovine serum (PAN, Aidenbach, Cat. #2602‐P130707), penicillin‐streptomycin (Gibco, Cat. #15140163), 0.1 mmol/L β‐mercaptoethanol and 1000 U/mL mouse leukaemia inhibitory factor. C2C12, Hepa1‐6 and CT26 were cultured in DMEM/F‐12 (Invitrogen, Cat. #C11330500BT) with 10% foetal bovine serum (Gemini, Cat. #900‐108), penicillin‐streptomycin (Gibco, Cat. #15140163) and 0.1 mmol/L β‐mercaptoethanol. For EB differentiation, mESCs were cultured in ultra‐low adherent plates. mESC medium without LIF (DMEM with non‐essential amino acids, L‐glutamine, foetal bovine serum, penicillin‐streptomycin and β‐mercaptoethanol) was used. For ESC differentiation without LIF, ESC medium without LIF was used. For mESC differentiation induced by retinoic acid (RA), 1 µmol/L RA was added to mESC medium without LIF.

### 2.2 C2C12 differentiation

C2C12 was cultured in differentiation medium containing DMEM/F12 (Invitrogen, Cat. #C11330500BT), 100 mg/L BSA (MP Biomedicals, Cat. #02180728.1), 4 mg/L transferrin, 30 nmol/L sodium selenite, 1 mg/L α‐tocopherol, 0.1 mmol/L β‐mercaptoethanol, 0.5 mg/L ethanolamine, 0.5 mg/L insulin and penicillin‐streptomycin.

### 2.3 In‐house KLF3 antibody

Antigen KLF3 (14‐231AA) with C‐terminal His‐tag was extracted in the presence of 5 mol/L urea and 0.1% SDS from *E coli* BL21(DE3) plysS. Purification was made by HisPur Cobalt Resin (Invitrogen). The KLF3 rabbit polyclonal antibody was made in Biodragon Immunotech Company.

### 2.4 Western blot

Cells were collected and directly lysed in lysis buffer containing RIPA Buffer (Beyotime) with PMSF (Sigma) and phosphatase inhibitor (Roche). Tissues were washed by cold PBS and then homogenized by IKA T10 homogenizer in lysis buffer. Proteins were quantified using Pierce BCA Protein Assay Kit (Thermo Scientific). Equal amounts of proteins were loaded for immunoblotting. Proteins were electroblotted to PVDF membranes; then, PBS with QuickBlot (Beyotime) was used to block membranes. Antibodies used were rabbit anti‐KLF3 (in‐house), goat anti‐KLF3 (Abnova, Cat. #PAB6147), mouse anti‐GAPDH (Beyotime, Cat. #AF0006), mouse anti‐β‐ACTIN (Biodragon, Cat. #B1029), mouse anti‐β‐TUBULIN (Biodragon, Cat. #B1031), mouse HSP90β (Beyotime, Cat. # AF0192), rabbit anti‐CKM (ProteinTech, Cat. #18712‐1‐AP) and rabbit anti‐MYL1 (ProteinTech, Cat. # 15814‐1‐AP). Uncropped Western blotting images are provided in Figure S7.

### 2.5 RNA extraction, reverse transcription and qPCR

Total RNA was extracted according to standard TRIzol protocol (Invitrogen, Cat. #15596026) and was quantified by Biodropsis BD2000 (OSTC). Isolated RNA was reverse‐transcribed into complementary DNA (cDNA) using the HiScript II QRT SuperMix kit (Vazyme, Cat. #R223). Real‐time PCR was performed on Step One Plus Real‐Time PCR System (Applied Biosystems), and AceQ qPCR SYBR Green Master Mix (Vazyme, Cat. #Q141) was used for gene amplification and quantitation. Primers are listed in Table S3. Source data for qPCR analysis are provided in Data S1.

### 2.6 Polysome fractionation assay

Cells were treated with 100 μg/mL cycloheximide for 5 minutes and then scraped with ice‐cold PBS containing 100 μg/mL cycloheximide, protease inhibitor (Thermo Scientific, Cat. #A32965) and RNase inhibitor (Ambion, Cat. #AM2684). Pellet cells at 3000 *g* for 5 minutes then re‐suspend them in ice‐cold lysis buffer containing 30 mmol/L Tris‐Hcl pH8.0, 150 mmol/L NaCl, 1% Triton X‐100, 5 mmol/L MgCl_2,_ 1 mmol/L DTT, protease inhibitor, RNase inhibitor and 200 μg/mL cycloheximide. Cells were lysed at 4°C for 30 minutes and then centrifuged at 3000 *g* for 5 minutes. Lysate on the supernatant was layered on the top of 10%‐45% sucrose gradients (20 mmol/L Hepes‐KOH pH7.6, 100 mmol/L KCl, 15 mmol/L MgCl2, 1 mmol/L DTT, protease inhibitor, RNase inhibitor and 200 μg/mL cycloheximide), which is made by Gradient Master (Biocomp Instruments). Gradients were centrifuged at 4°C for 3 hours at 35 000 RPM in a SW‐41 rotor, and 12 fractions were then collected using Piston Gradient Fractionator (Biocomp Instruments) and Bio‐Rad Econo System (Bio‐Rad Laboratories). Before the extraction of RNA from each fraction, tagRFP mRNA was added as spike‐in. For qPCR data analysis, the spike‐in RFP mRNA was used as control.

### 2.7 IF staining

Cells were fixed with 4% paraformaldehyde for 20 minutes at room temperature. After the fixation, cells were permeabilized with 0.25% Triton X‐100 for 20 minutes at room temperature and blocked with 3% FBS in PBS for 1 hour at room temperature. Cells were then incubated with primary antibodies (1:200, anti‐KLF3, Abnova, Cat. #PAB6147) diluted in PBS with 3% FBS for 2 hours. After washing three times with PBS, the cells were incubated with secondary antibody (1:200, anti‐Goat IgG Alexa Fluor 488) for 1 hour and followed by DAPI staining. For preimplantation embryos’ IF staining, 0.1% Tween‐20 was added in PBS.

### 2.8 Vector construction

Doxycycline‐inducible plasmids were constructed from pBlueScript II; PciI and PsiI were used to remove unnecessary sequence. Plasmids expressing various fusion proteins of KLF3 and Renilla were modified from psiCheck‐2 (Promega). Codon optimization of Klf3 was performed by the software at https://www.genscript.com/tools/rare-codon-analysis. Codon optimized Klf3 and proline‐mutated Klf3 sequences were synthesized by GENEWIZ Company.

### 2.9 Luciferase assay

Cells were seeded in 96‐well plates and grown for 16 hours, and then transfected with plasmids and cultured for another 48 hours before lysis. Protein were extracted and processed for luciferase assay using the Dual‐Luciferase Reporter Assay System (Promega). Source data for luciferase assay are provided in Data S1.

### 2.10 Construction of Klf3 reporter cell line and Klf3 overexpressing cell line

For the generation of KLF3‐GFP reporter cell line, the N terminal of KLF3 without DNA‐binding domain was used. The PB‐CAGGS‐Klf3‐GFP‐EF1α‐DsRed construct was transfected into mESCs and selected with hygromycin B for 5d. Colonies containing DsRed fluorescence were then picked and expanded. For the inducible expression of KLF3 in mESCs, R26 cell line which contained Col1A1 FRT site and R26‐M2rtTA was used.^27^ Invitrogen NEON Transfection System was used to transfect pFT‐Klf3 or pFT‐GFP and pCAGGS‐flpE (Addgene plasmid #20733) into R26 cells. Transfected cells were seeded on feeder cells and selected by hygromycin B. Clones with correct insertion of Klf3 or GFP at FRT site were then picked for further experiments. Doxycycline was used at ~1 µmol/L for the induction of gene expression.

### 2.11 Flow cytometry

Cells were trypsinized and re‐suspended in ice‐cold PBS containing 2% FBS. Sorting was performed using Aria SORP (Becton, Dickinson and Company). During sorting, cells were collected in culture medium and kept at 4°C. The fraction of KLF3‐GFP‐positive cells was analysed by BD LSRFortessa SORP (Becton, Dickinson and Company). Data were analysed by FlowJo software.

### 2.12 Low‐input RT‐qPCR

For each sample, 10 cells were sorted by flow cytometry into 2 μL mild hypotonic lysis buffer composed of 0.2% Triton X‐100 and 2 U/μL of RNase inhibitor (Ambion, Cat. #AM2684). Reverse transcription reaction and PCR pre‐amplification were performed as previously described.^12^, ^28^ The qPCR was performed using AceQ qPCR SYBR Green Mater Mix (Vazyme, Cat. #Q141).

### 2.13 RNA‐seq and bioinformatics analysis

RNA was extracted from Klf3‐OE and GFP‐OE cells; then, they were enriched twice with poly‐T oligo‐attached magnetic beads and then subjected to the synthesis of double‐stranded (ds) cDNA. The ds‐cDNA was constructed with NEBNext Ultra Directional RNA Library Prep Kit (NEB cat. #E7420S) and sequenced by Illumina HiSeq platform (Novogene, Tianjin, China). For low‐input KLF3‐GFP‐positive and KLF3‐GFP‐negative cells, 500 cells were sorted by flow cytometry into mild hypotonic lysis buffer and Samrt‐seq2 protocol was applied to construct RNA libraries.^28^ The RNA‐seq libraries were sequenced on Illumina HiSeq platform (Genewiz). All sequencing reads were aligned to the mouse genome (mm10) with STAR (version 2.5.0) using the GENCODE transcript annotation as transcriptome guide. All programs were processed following default settings except for special annotation. The FPKM values generated by Cufflinks (version 2.2.1) were used to quantify the expression level. Differentially expressed genes were determined by DESeq2. GO enrichment analysis was differentially expressed genes with fold change > 2 or < 0.5 and *P*‐value < .01, and was performed with DAVID v.6.8. The enrichment of selected gene sets was calculated by java GSEA Desktop Application. R software (v3.5.1) was used for the generation of scatter plot, box plot and Venn plot. The 2C‐specific ZGA genes are genes activated during ZGA (the 2C stage) that are also enriched in 2C::tdTomato^+^ cells.^7^ Single cell RNA‐seq data from Ref 39 were used to define 8‐cell‐embryo activated genes, which are upregulated at least 16‐fold from 4‐cell to 8‐cell stage. For KLF3 motif enrichment analysis, KLF3 binding motif was obtained from MEME suite (http://meme-suite.org/db/motifs). The promoter region was defined as −1 to + 1kb around transcription start sites. RNA‐sequencing data have been deposited in the Gene Expression Omnibus (GEO) under accession code GSE157790.

### 2.14 Quantification and statistical analysis

Data were presented as mean ± SD except where indicated otherwise. Statistical analyses were performed using the GraphPad Prism v6 software and R software (v3.5.1). Statistical significance was assessed by two‐tailed *t* test. For multiple comparison, the *P*‐value was calculated by one‐way or two‐way ANOVA with Dunnett's test.

## 3 RESULTS

### 3.1 The Klf3 mRNA but not protein is expressed in mESCs

**FIGURE 1:**
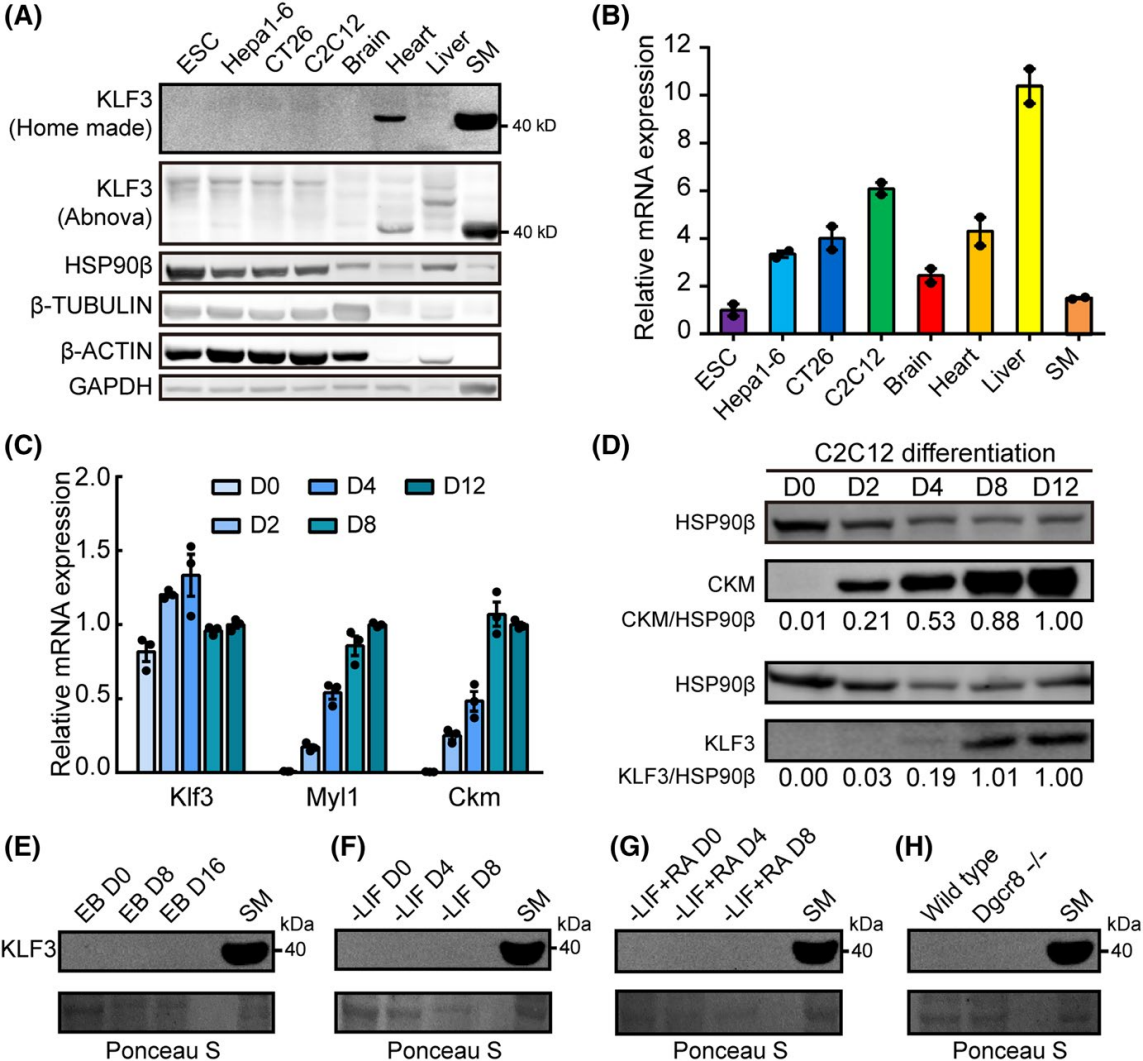
The expression of KLF3 protein is repressed in mESCs. A, Western blotting analysis of KLF3 protein in wild‐type mESCs (V6.5), Hepa1‐6, CT26, brain, heart, liver and skeletal muscle. Two antibodies were shown including in‐house antibody and commercial antibody from Abnova. For each sample, 20 μg protein was loaded. B, Relative RNA expression of Klf3 gene in wild‐type mESCs (V6.5), Hepa1‐6, CT26, brain, heart, liver and skeletal muscle. The Hsp90ab1 gene was used as a control. For each gene, data were normalized to the mRNA level of wild‐type mESCs (V6.5). Shown is mean with range of two biological replicates. C, Relative RNA expression of Klf3, Ckm and Myl1 during C2C12 differentiation. The Gapdh gene was used as a control. For each gene, data were normalized to the mRNA level in day 12 differentiated C2C12. Shown is mean ± SD, n = 3 biological replicates. D, Western blotting analysis of KLF3 and CKM during C2C12 differentiation. For each sample, 20 μg protein was loaded. Data were quantification of protein level normalized to HSP90β and then to day 12 differentiated C2C12. Results from another independent experiment are shown in Figure S1B. E‐H, Western blotting analysis of KLF3 protein level in embryoid body (E), differentiated mESCs in media without LIF (F), differentiated ESCs induced by RA (G) and *Dgcr8* knockout mESCs (H). For each sample, 20 μg protein was loaded. An empty lane was added between SM and other samples to avoid cross contamination. Ponceau S was used as input control

We first analysed the expression of Klf family TFs in mESCs based on previously published RNA‐seq in mESCs^29^, ^30^, ^31^, ^32^, ^33^, ^34^ (Figure S1A). As expected, pluripotency regulating Klf2, Klf4 and Klf5 is expressed at high levels in mESCs. Interestingly, we found that Klf3, Klf9, Klf10 and Klf16 are also expressed at relatively high levels, although lower than these canonical pluripotency regulating KLF genes. Previous studies have reported that Klf3 plays important roles in erythropoiesis,^35^ adipogenesis,^36^ lymphopoiesis^37^ and cardiovascular development.^38^ We then focused our analysis on Klf3. We first checked the protein expression in V6.5 mESCs with commercial and in‐house antibodies. Surprisingly, KLF3 protein was barely detectable in V6.5 mESCs (Figure 1A). In contrast, in skeletal muscle (SM) which expressed mRNA at similar levels as mESCs, KLF3 protein was highly expressed. We further checked KLF3 protein level in Hepa1‐6, CT26, C2C12, brain, liver and heart. Interestingly, even though Klf3 mRNA was found to be expressed at higher levels in these samples than in SM, only heart expressed detectable KLF3 protein (Figure 1A,B). These results demonstrate that Klf3 protein is repressed in mESCs and various other cells.

### 3.2 The inhibition of KLF3 protein expression is relieved in differentiating myoblasts but not mESCs

C2C12 can be differentiated into SM lineage in vitro. We then checked whether KLF3 protein becomes expressed during C2C12 differentiation. Consistent with successful differentiation into SM lineage, SM markers Ckm and Myl1 were gradually but strongly upregulated at both RNA and protein levels (Figure 1C,D). Interestingly, although Klf3 mRNA was not significantly varied at different time points, KLF3 protein was extensively upregulated during SM differentiation (Figures 1C,D and S1B). Next we checked whether KLF3 protein level is upregulated during mESC differentiation. We failed to detect any KLF3 protein in embryoid bodies, mESCs differentiated in media without LIF supplement or mESCs differentiated in the presence of all trans‐retinoid acid (RA) (Figure 1E‐G and S1C), suggesting that KLF3 protein is also repressed in differentiating mESCs. Likewise, we also failed to detect KLF3 protein in a *Dgcr8* knockout mESC line (Figure 1H and S1C), indicating that the repression of KLF3 protein was not due to miRNA‐mediated regulation. Together, these results demonstrate that the expression of KLF3 protein is suppressed in many different cells but can be dynamically upregulated during myoblast differentiation.

### 3.3 KLF3 protein is translated but repressed through degradation in mESCs

**FIGURE 2:**
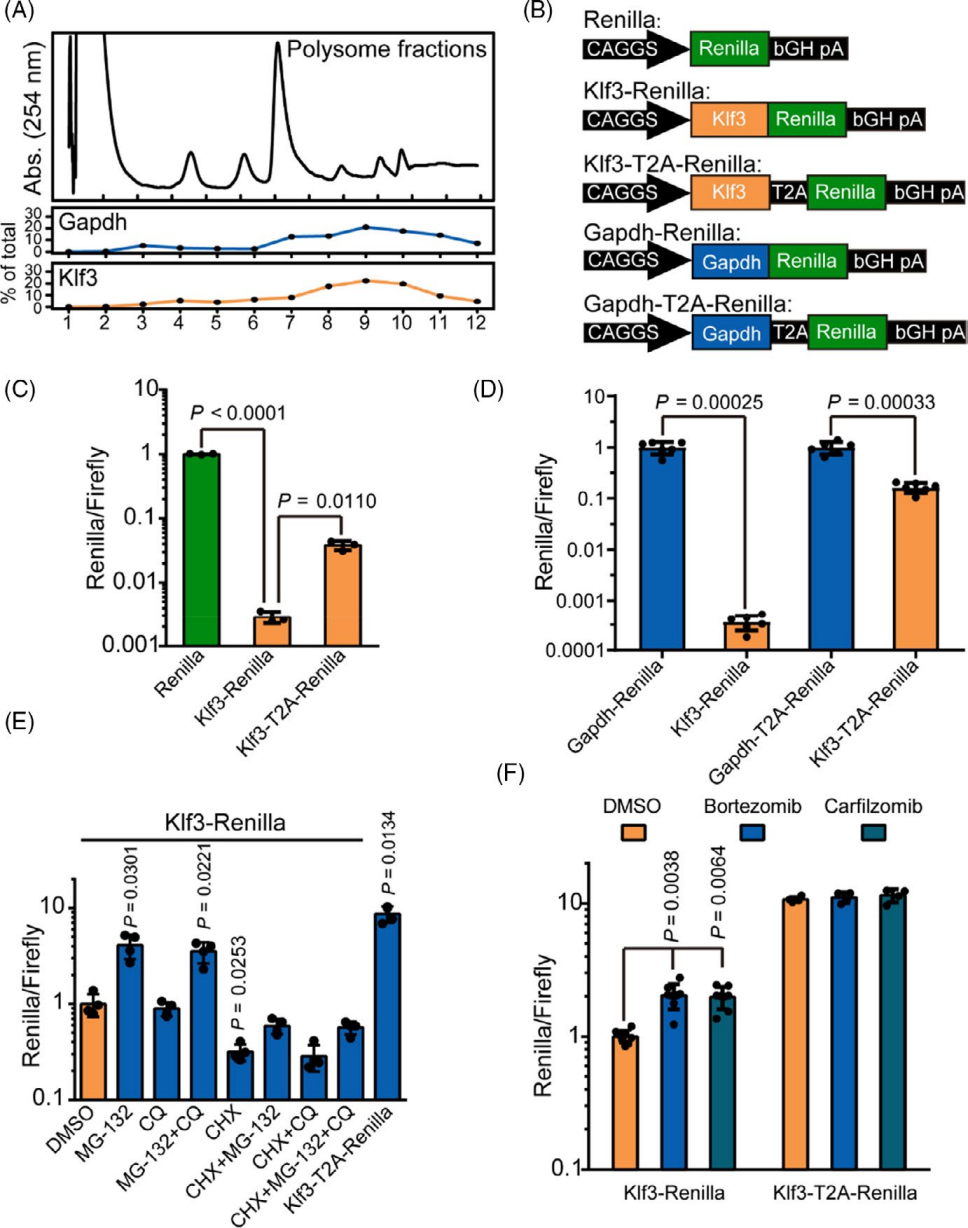
KLF3 protein is translated but degraded in mESCs. A, Ribosome profiling of mESCs. Up, monitoring of absorbance at 254 nm for 12 fractions collected in polysome fractionation assay; lower two panels are percentage of Gapdh and Klf3 in each fraction. For each gene, data were normalized to spike‐in mRNA. B, Schematic representation of the structure of Klf3 gene in psiCheck‐2 plasmid used for luciferase reporter assay. C, Luciferase reporter assay of mESCs transfected with Renilla, Klf3‐Renilla and Klf3‐T2A‐Renilla. For each construct, data were normalized to firefly control and then to Renilla construct. Shown is mean ± SD, n = 3. The *P* value was calculated by unpaired one‐way ANOVA with two‐sided Dunnett's test compared with Klf3‐Renilla. D, Luciferase reporter assay of mESCs transfected with Gapdh‐Renilla, Klf3‐Renilla, Gapdh‐T2A‐Renilla and Klf3‐T2A‐Renilla. Data were normalized to firefly control then to respective Gapdh construct. Shown is mean ± SD, n = 6. The *P* value was calculated by unpaired two‐tailed Student's *t* test. E, Luciferase reporter assay of mESCs transfected with KLF3‐Renilla and treated with MG‐132, CQ and CHX. For each sample, data were normalized to firefly control and then to KLF3‐Renilla treated with DMSO control. Shown is mean ± SD, n = 4. The *P* value was determined by unpaired one‐way ANOVA with two‐sided Dunnett's test compared with KLF3‐Renilla treated with DMSO control. F, Luciferase reporter assay of mESCs transfected with KLF3‐Renilla and KLF3‐T2A‐Renilla and treated with bortezomib and carfilzomib. For each sample, data were normalized to firefly control and then to KLF3‐Renilla treated with DMSO control. Shown is mean ± SD, for KLF3‐Renilla, n = 8, for KLF3‐T2A‐Renilla, n = 4. The *P* value was determined by unpaired two‐way ANOVA with two‐sided Dunnett's test compared with KLF3‐Renilla treated with DMSO control

Next we sought to understand mechanism underlying KLF3 protein repression in mESCs and C2C12. The repression of protein expression could happen at translational or post‐translational level. We first performed polysome fractionation assay to check whether Klf3 mRNA is translated. The results showed that Klf3 mRNA was bound by polysome in a similar pattern to highly translated Gapdh mRNA (Figure 2A), suggesting that Klf3 is translated in ESCs. Like in ESCs, Klf3 was also bound by polysome in C2C12 cells, and there was no difference in the binding pattern between C2C12 cells and differentiated C2C12 cells (Figure S2). These data suggest that KLF3 protein is translated but repressed through post‐translational regulation in mESCs and C2C12.

Since KLF3 protein was successfully translated, we then checked whether KLF3 protein is degraded in mESCs. We tested this possibility through a sensitive assay using KLF3 and Renilla luciferase fusion constructs (Figure 2B). Compared to unfused Renilla control, Klf3‐Renilla was repressed around 500‐fold in mESCs (Figure 2C). This result confirmed the repression of KLF3 protein in mESCs. To distinguish degradation versus translational repression, we introduced T2A peptide sequence between Klf3 and Renilla luciferase (Figure 2B). Because T2A peptide can induce ‘ribosome skipping’ between last 2 amino acids,^39^ KLF3 and Renilla will be produced as two independent proteins, therefore excluding destabilization effects due to KLF3 peptide sequence on Renilla. In other words, if KLF3 is repressed through degradation, the split between KLF3 and Renilla will likely lead to higher luciferase expression. Indeed, we observed ~10‐fold increase in Renilla level upon the introduction of T2A peptide (Figure 2C). We further analysed the expression difference of Renilla between Gapdh‐Renilla and Klf3‐Renilla constructs, and between Gapdh‐T2A‐Renilla and Klf3‐T2A‐Renilla constructs (Figure 2D). Theoretically, the difference between GAPDH‐Renilla and KLF3‐Renilla will reflect both degradation and translational repression, while the difference between GAPDH‐T2A‐Renilla and KLF3‐T2A‐Renilla will only reflect translational repression since Renilla protein is detached from KLF3 or GAPDH during translation process. While KLF3‐Renilla activity is ~2800‐fold lower than GAPDH‐Renilla, KLF3‐T2A‐Renilla activity is only 6‐fold lower than GAPDH‐T2A‐Renilla (Figure 2D). These data suggest that degradation may be the major pathway inhibiting KLF3 protein expression in mESCs, but translational repression could also play a role. Next we tested through which pathway KLF3 is degraded in ESCs. Proteins are usually degraded through the proteasome pathway or lysosome pathway, and these two pathways can be inhibited by MG‐132 and chloroquine (CQ), respectively. Adding MG132 but not CQ led to ~3‐fold increase in KLF3‐Renilla level (Figure 2E). Moreover, when the protein synthesis was inhibited by the treatment of cycloheximide (CHX), KLF3‐Renilla was further decreased. However, MG132 significantly rescued KLF3‐Renilla level in the presence of CHX, supporting KLF3 being degraded by proteasome pathway (Figure 2E). Consistently, two other proteasome inhibitors bortezomib and carfilzomib also significantly increased KLF3‐Renilla activity (Figure 2F). In contrast, none of them increased Renilla activity in KLF3‐T2A‐Renilla construct (Figure 2F). Together, these data show that KLF3 protein is translated but degraded through proteasome pathway. However, our data do not exclude the role of other mechanisms such as translational repression in inhibiting KLF3 protein expression.

### 3.4 The proline‐rich sequence promotes the degradation of KLF3

**FIGURE 3:**
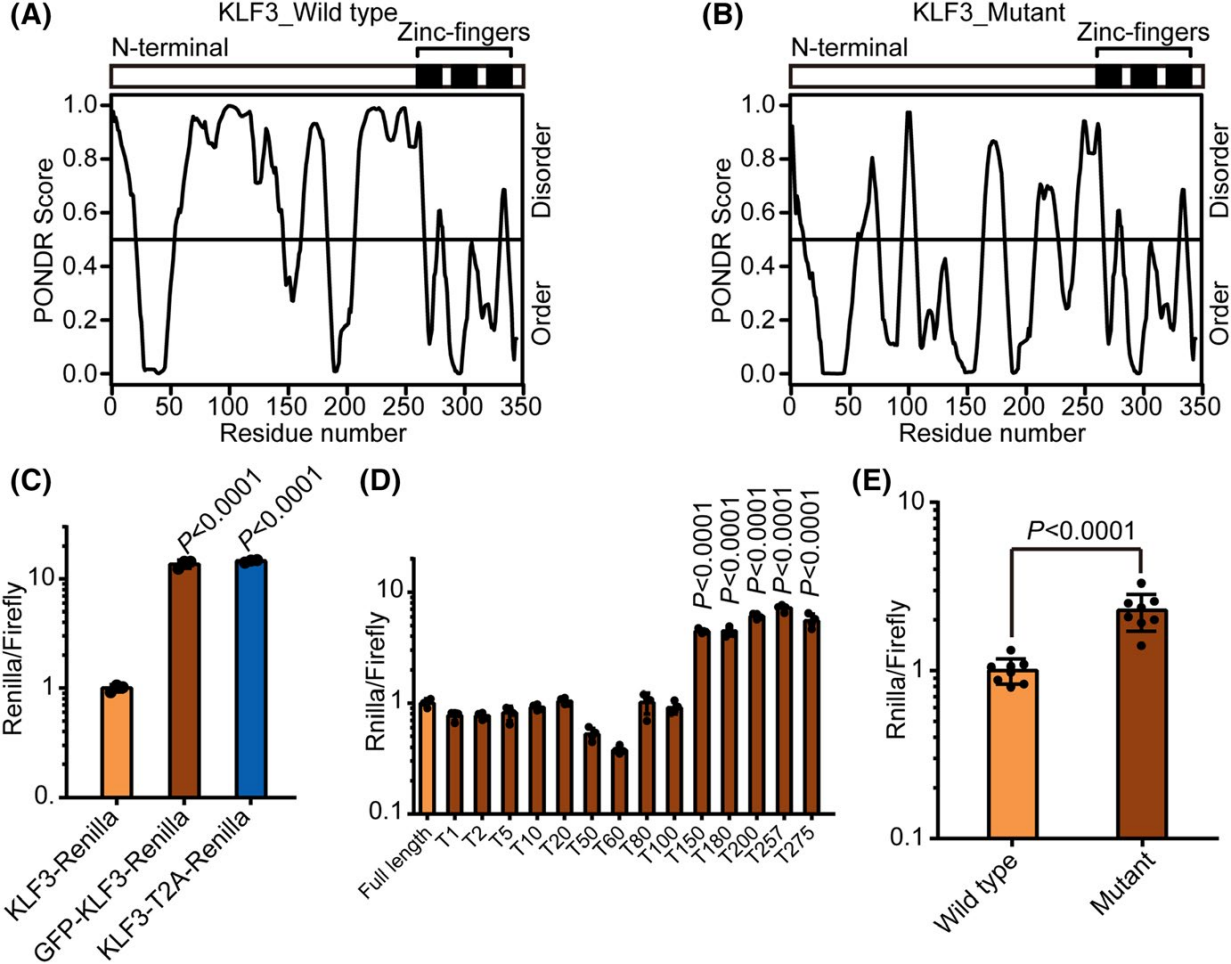
Intrinsically disordered structure promotes the degradation of KLF3 protein in mESCs. A‐B, PONDR score of KLF3 (A) and proline‐mutated KLF3 (B) predicted by PONDR program. C, Luciferase reporter assay of ESCs transfected with KLF3‐Renilla, GFP‐KLF3‐Renilla and KLF3‐T2A‐Renilla. For each sample, data were normalized to firefly control and then to KLF3‐Renilla. Shown is mean ± SD, n = 3. The *P* value was determined by unpaired one‐way ANOVA with two‐sided Dunnett's test compared with KLF3‐Renilla. D, Luciferase reporter assay of mESCs transfected with N‐terminal truncated KLF3‐Renilla. Number of truncated amino acids at the N terminal is indicated. For each sample, data were normalized to firefly and then to full‐length KLF3‐Renilla. Shown is mean ± SD, n = 4. The *P* value was determined by unpaired one‐way ANOVA with two‐sided Dunnett's test compared with full‐length KLF3‐Renilla. E, Luciferase reporter assay of mESCs transfected with wild‐type KLF3‐Renilla and proline‐mutated KLF3‐Renilla. For each sample, data were normalized to firefly and then to Klf3‐Renilla. Shown is mean ± SD, n = 8. The *P* value was determined by two‐tailed Student's *t* test

To gain further insights on why KLF3 is degraded in mESCs, we predicted disorder structure in KLF3 through PONDR program.^40^ Interesting, we found that N terminal of KLF3 was highly unstructured (Figure 3A). Proline often causes disordered structure due to its constrained conformation. We noticed that there were 46 prolines in N terminal of KLF3 protein (Figure S3). When these prolines were mutated to alanine, the proportion of disordered structure was largely reduced (Figure 3B). We then experimentally tested the hypothesis that disordered structure in N terminal of KLF3 leads to its degradation in mESCs. Three lines of evidence supported the hypothesis. First, fusing a stable protein GFP to the N terminal of KLF3‐Renilla significantly increased the Renilla activity (Figure 3C); second, truncation of 150 amino acids or more at KLF3 N terminal significantly increased the expression of KLF3‐Renilla (Figure 3D); finally, mutation of all prolines to alanines in the N terminal also significantly increased the expression of KLF3‐Renilla (Figure 3E). These data suggest that disordered structure of KLF3 at the N terminal made it vulnerable to degradation in mESCs.

### 3.5 KLF3 protein is expressed in 8‐cell stage mouse embryos

**FIGURE 4:**
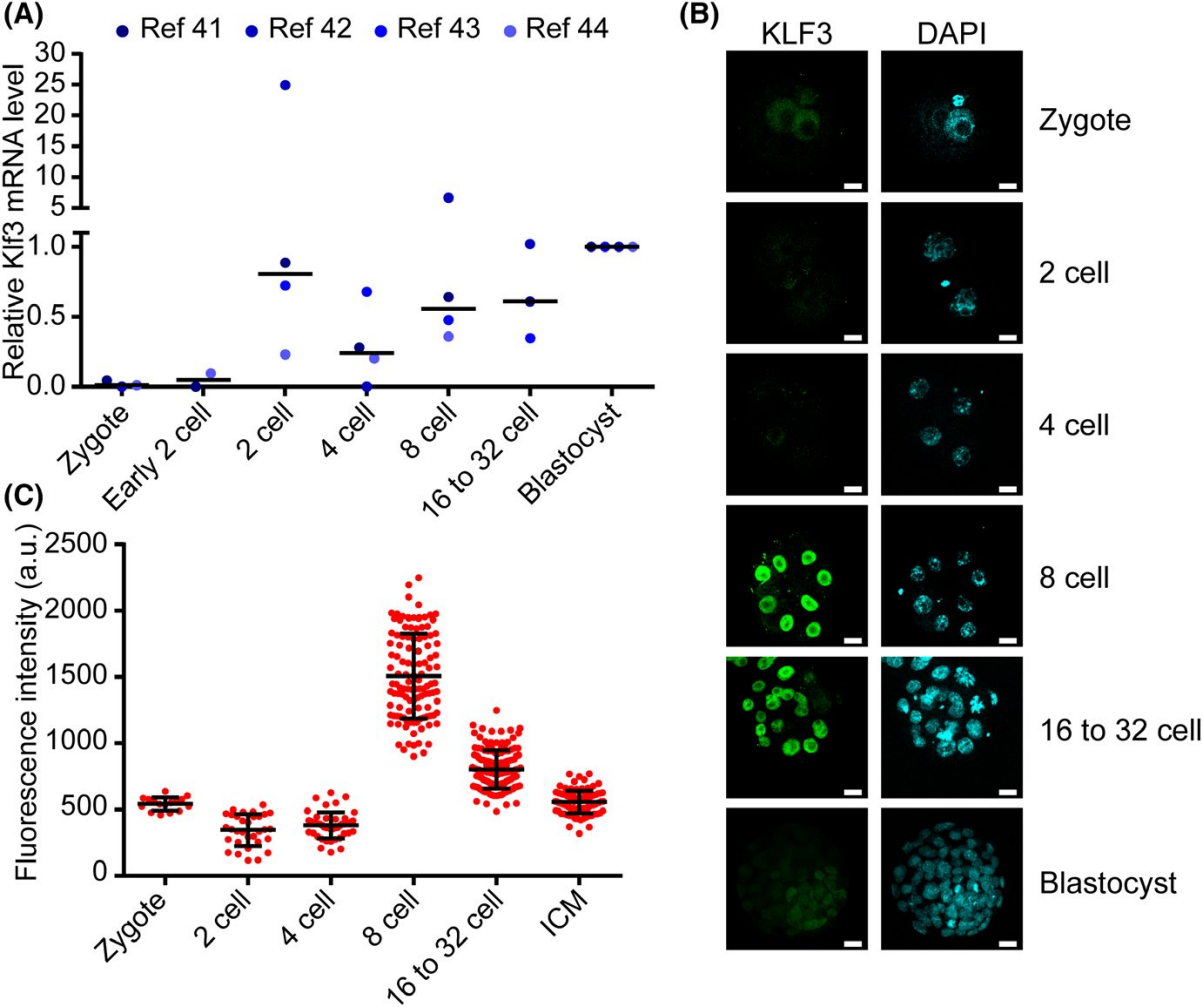
KLF3 protein is highly expressed in 8‐cell embryos. A, Relative expression level of Klf3 from zygote to blastocyst. For each stage, FPKM were normalized to blastocyst. Centre line, median. B, IF staining of KLF3 protein in mouse embryos from zygote to blastocyst stage. Commercial antibody anti‐KLF3 from Abnova was used. Shown are representative images. Scale bars, 20 μm. C, Quantification of fluorescence intensity in nucleus. Shown is mean ± SD. Each dot represents one nucleus. n = 8 to 16 embryos for each stage

Next we checked whether KLF3 protein expression is dynamically regulated during early embryo development from zygote to blastocyst stage. Analysis of previously published single cell RNA‐seq data^41^, ^42^, ^43^, ^44^ indicates that Klf3 mRNA was activated at 2‐cell stage, and remained at high level until blastocyst stage (Figure 4A). To check KLF3 protein level, we performed immunofluorescence staining (IF) for KLF3 protein from fertilized egg to blastocyst stage. Interestingly, we found that KLF3 protein is barely detectable before or after 8‐cell stage (Figure 4B,C). However, KLF3 protein was detected at high levels at 8‐cell stage embryo (Figure 4B,C). These data suggest that KLF3 protein is post‐translationally regulated in preimplantation mouse embryos with highest expression at 8‐cell stage.

### 3.6 KLF3 protein is expressed in a small subset of mESCs

**FIGURE 5:**
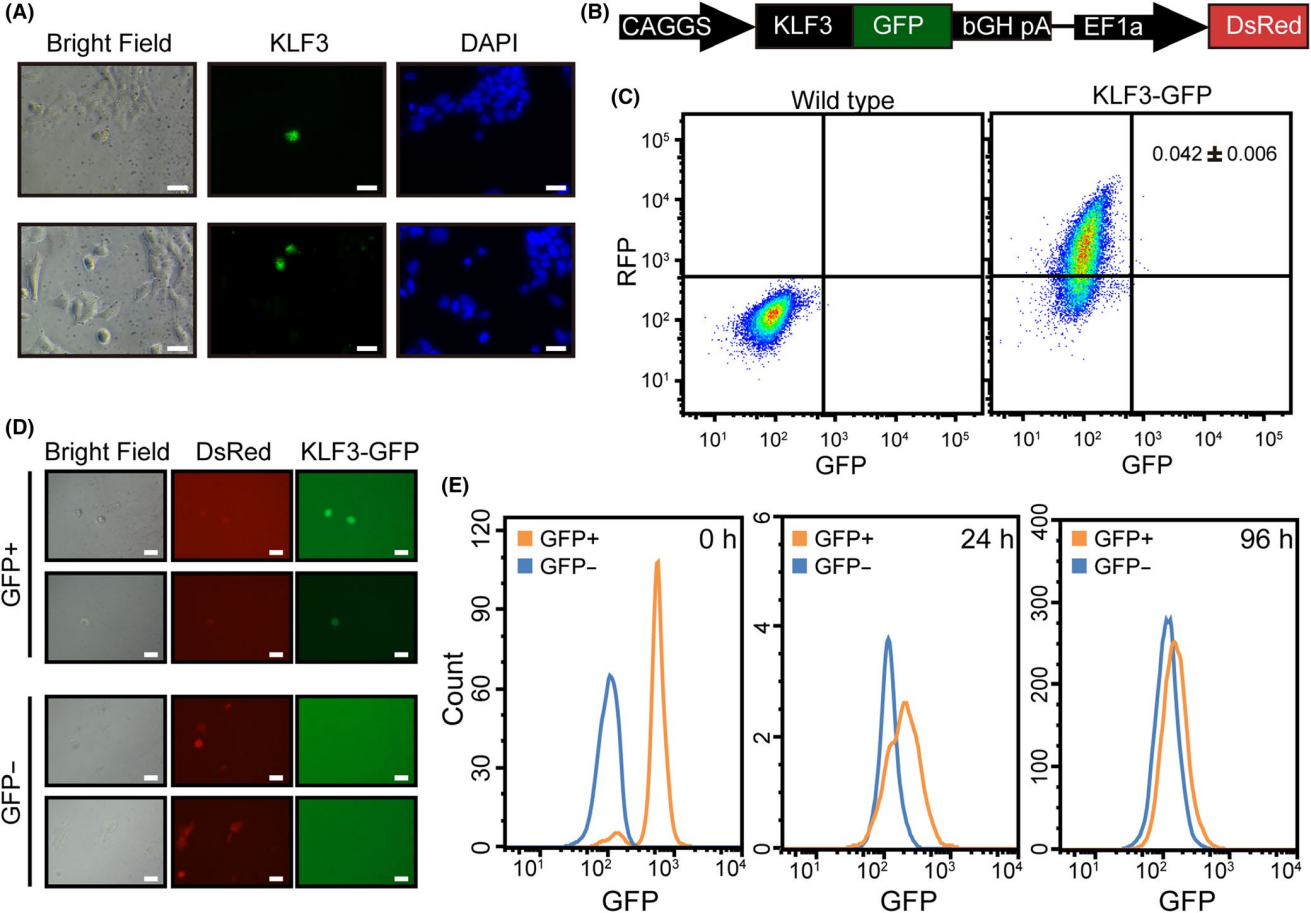
KLF3 protein is expressed in a small subset of mESCs. A, IF staining of KLF3 in mESC culture. Left panel, bright field images; middle panel, fluorescence of KLF3 antibody staining; right, fluorescence of DAPI images. Two areas are shown. Scale bars, 20 μm. B, Schematic representation of the structure of KLF3‐GFP fusion protein. C, Flow cytometry analysis of KLF3‐GFP‐positive mESCs. Shown is percentage of GFP‐positive cells. Data are shown as mean ± SD, n = 3. D, Microscopic images of KLF3‐GFP‐positive and KLF3‐GFP‐negative cells cultured 24 h after sorting. Left, bright field images; middle, DsRed; right, GFP. Scale bars, 20 μm. E, Histogram of KLF3‐GFP‐positive and KLF3‐GFP‐negative mESCs. Shown are time points of 0, 24 and 96 h after sorting

Previously, a rare population of cells in ESC culture was found to mimic 2‐cell state, specifically on the expression of 2‐cell specific genes including murine endogenous retroviruses (MERVL) and Zscan4.^7^ Inspired by this study, we wondered whether there are any mESCs mimicking 8‐cell embryos in terms of KLF3 protein expression. To check this possibility, we performed IF for KLF3 in mESCs. We found that KLF3 protein can indeed be detected in a small subset of mESCs (Figure 5A). To verify this finding, we made a mESC line which expresses an Emerald GFP protein fused to the C terminal of KLF3 (Figure 5B). Flow cytometry analysis showed that around 0.04% ESCs are GFP‐positive (Figure 5C). We then sorted the GFP‐positive cells out using fluorescence activated cell sorting instrument and cultured them in mESC media. The fraction of GFP‐positive cells was quickly decreased and almost all cells became GFP‐negative after 96 hours (Figure 5D‐E). These data indicate that the repression of KLF3 protein is relieved in a small subset of mESCs.

### 3.7 The transcriptome difference between KLF3‐positive and KLF3‐negative mESCs

**FIGURE 6:**
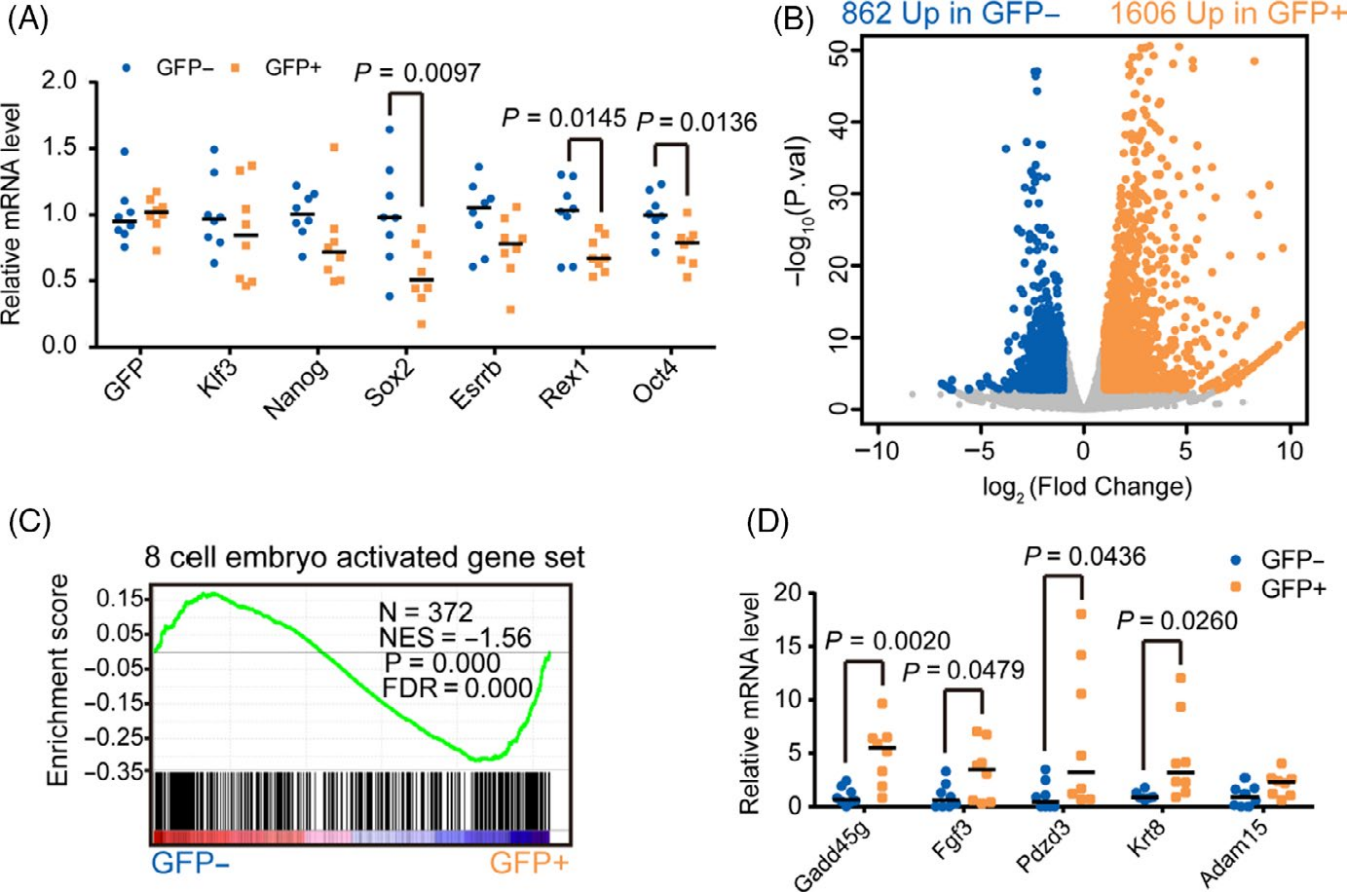
KLF3‐GFP‐positive mESCs enrich 8‐cell‐embryo activated genes. A, RT‐qPCR of GFP, Klf3 and pluripotency genes in KLF3‐GFP‐positive and KLF3‐GFP‐negative cells. The β‐actin gene was used as a control. For each gene, data were normalized to the average mRNA level of KLF3‐GFP‐negative cells. Centre line, median, n = 8. The *P* value was calculated by two‐tailed Student's *t* test. B, Volcano plot showing gene expression difference in KLF3‐GFP‐positive and KLF3‐GFP‐negative mESCs. Yellow, upregulated genes in KLF3‐GFP‐positive cells. Blue, upregulated genes in KLF3‐GFP‐negative cells. C, GSEA for 8‐cell‐embryo activated genes in KLF3‐GFP‐positive and KLF3‐GFP‐negative cells. For x‐axis, genes were ranked based on the ratio of KLF3‐GFP‐negative versus positive mESCs. D, RT‐qPCR of representative 8‐cell‐embryo activated genes in KLF3‐GFP‐positive and KLF3‐GFP‐negative cells. The β‐actin gene was used as a control. For each gene, data were normalized to the average mRNA level of KLF3‐GFP‐negative cells. Centre line, median, n = 8. The *P* value was calculated by two‐tailed Student's *t* test

We then performed qPCR analysis for Klf3 and key pluripotency genes in KLF3‐GFP‐positive mESCs. Consistent with regulation at protein level, Klf3 mRNA was similar in GFP‐positive and GFP‐negative cells (Figure 6A). Moreover, pluripotency genes including Nanog, Oct4, Esrrb, Rex1 and Sox2 were slightly repressed in GFP‐positive cells (Figure 6A). To fully characterize KLF3‐GFP‐positive cells, we performed RNA‐seq. Overall, there were 862 and 1606 genes that are downregulated and upregulated in GFP‐positive versus GFP‐negative mESCs (Figure 6B and Table S1), respectively. These differentially regulated genes were enriched in lysosomal, protein processing and metabolic pathways (Figure S4A,B). In addition, analysis of 2‐cell specific genes indicates that these rare KLF3‐GFP‐positive mESCs are different from previously identified 2C like cells (Figure S4C,D). Moreover, RNA‐seq analysis indicated that KLF3‐GFP‐positive mESCs are different from previously reported Zscan4::GFP‐positive,^45^ MERVL::tdTomato‐positive^46^ or Nanog::VNP‐negative^47^ mESCs (Figure S4E‐G). These results identify previously unknown cell populations in ESC culture with distinct transcriptome from the majority of mESCs.

### 3.8 KLF3‐GFP‐positive ESCs upregulate 8‐cell‐embryo activated genes

Since KLF3 protein is also highly expressed in 8‐cell embryos, we then checked the similarity in transcriptome of 8‐cell embryos and KLF3‐positive mESCs. GSEA analysis showed that genes activated from 4‐ to 8‐cell stage^41^ were significantly enriched in KLF3‐GFP‐positive mESCs (Figures 6C and S5A,B). RT‐qPCR analysis also confirmed the upregulation of selected 8‐cell‐embryo activated genes in KLF3‐GFP‐positive mESCs (Figures 6D and S5C). Taken together, these data show that KLF3‐GFP expression marks a small subset of mESCs which upregulates 8‐cell‐embryo activated genes.

### 3.9 Forced expression of KLF3 protein upregulates 8‐cell‐embryo activated genes

**FIGURE 7:**
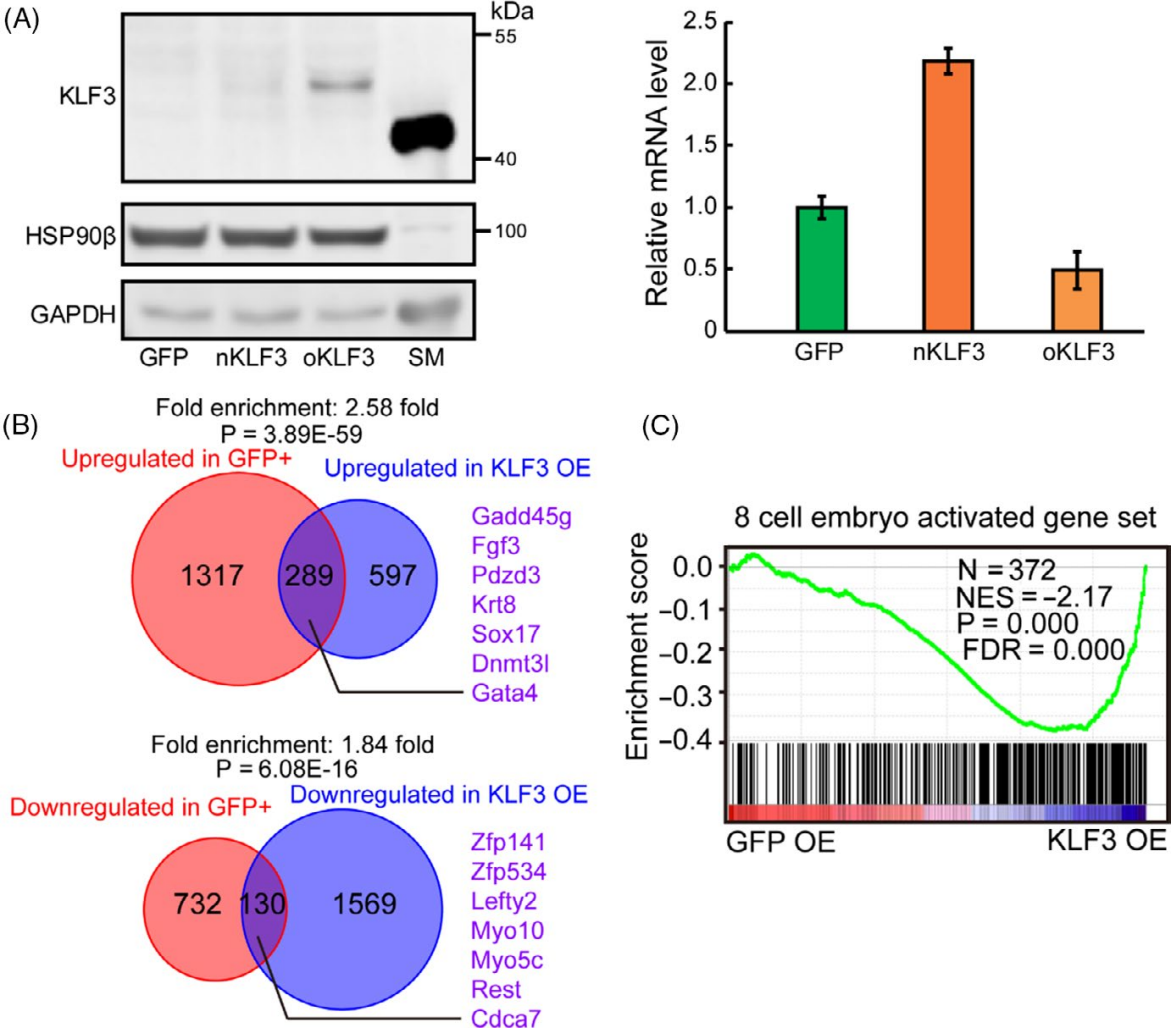
KLF3 overexpression upregulates 8‐cell‐embryo activated genes in mESCs. A, Overexpression of GFP, nKLF3 and oKLF3 in mESCs. Left panel, Western blotting analysis of KLF3 in GFP, nKLF3 and oKLF3 overexpressing mESCs. HSP90β and GAPDH were used as loading controls. Right panel, RT‐qPCR of expression level of exogenous genes in GFP, nKLF3 and oKLF3 overexpressing mESCs. The Gapdh mRNA was used as a control. Data were normalized to the average mRNA level of GFP overexpressing mESCs. Shown is mean ± SD, n = 3 biological replicates. B, The Venn diagram (up) shows the overlap between genes upregulated in KLF3‐GFP‐positive mESCs and genes upregulated in KLF3 overexpressing mESCs. The Venn diagram (bottom) shows the overlap between genes downregulated in KLF3‐GFP‐positive mESCs and genes downregulated in KLF3 overexpressing mESCs. Fold enrichment and *P* value are shown. The *P* value was calculated by hypergeometric test. C, GSEA for 8‐cell‐embryo activated genes in GFP and KLF3 overexpressing mESCs. For x‐axis, genes were ranked based on the ratio of GFP versus KLF3 overexpressing mESCs

The specific expression of KLF3 protein in 8‐cell embryos and the subset of mESCs suggest a potential function for KLF3 in these cells. To understand its impact on gene expression, we decided to overexpress KLF3 protein in mESCs. Expression of native Klf3 sequence did not lead to any detectable KLF3 protein in mESCs, consistent with the post‐translational regulation of KLF3 expression (Figure 7A). The steady level of a protein depends on both production and degradation rate. Since we lack means to specifically inhibit the degradation of KLF3, we reasoned that we may achieve higher KLF3 protein expression by increasing translation efficiency through codon optimization. Indeed, after codon optimization, we were able to detect trace amount of KLF3 protein in mESCs in a doxycycline‐inducible fashion. The amount of KLF3 protein in mESCs overexpressing codon optimized Klf3 (oKlf3) was around 15% as in skeletal muscle (Figure 7A). We noticed that overexpressed KLF3 protein migrated to a position of higher molecular weight than in skeletal muscle. Mass spectrometry analysis indicates that multiple serine of KLF3 was phosphorylated (Figure S6A). Treatment with λ protein phosphatase (λpp) shifted KLF3 protein back to the same migration rate in mESCs as in skeletal muscle (Figure S6B), indicating phosphorylation shifting KLF3 to higher molecular weight position during electrophoresis. To understand the impact of KLF3 on gene expression, we then performed RNA‐seq on mESCs overexpressing KLF3 and compared them with the transcriptome of KLF3‐GFP‐positive ESCs and 8‐cell embryos. Consistently, for both upregulated and downregulated genes, we observed significant overlap between KLF3 overexpressing and KLF3‐GFP‐positive mESCs (Figure 7B and Table S2). While the upregulated genes were enriched in pathways such as ECM‐receptor interaction and PI3K‐Akt signalling pathways in KEGG analysis (Figure S6C), the downregulated genes were not significantly enriched in any pathways. Moreover, as in KLF3‐GFP‐positive mESCs, genes activated from 4‐ to 8‐cell stage embryo were also significantly enriched in KLF3 overexpressing mESCs (Figures 7C and S6D). Importantly, analysis of promoter region of 8‐cell‐embryo activated genes found that 259 of 372 promoters contain canonical KLF3 binding motif (fold enrichment ~1.51, *P* value = 1.70E‐10). These results suggest that KLF3 protein may directly regulate some of 8‐cell‐embryo activated genes.

## 4 DISCUSSION

Previous studies have identified various different heterogeneous states in ESC cultures.^2^, ^3^, ^4^, ^5^ These states are often marked by the activity of specific enhancers or promoters such as those of *Nanog*, *Rex1* and *Stella*. Whether heterogeneous state due to other layers of regulation such as splicing activity or protein stability exists is an open question. Here we identify an extremely small subset of cells that can be discerned from the majority of mESCs based on post‐translational control of KLF3 protein expression. We find that Klf3 mRNA is expressed and translated in mESCs. However, KLF3 protein is degraded through proteasome pathway due to its intrinsically disordered structure. More interestingly, transcriptome analysis shows that KLF3‐GFP‐positive mESCs upregulate a significant fraction of transcripts that are activated during 4‐ to 8‐cell stage transition. KLF3 protein likely plays a functional role in regulating the transcription of these genes, since overexpression of KLF3 protein also leads to the upregulation of transcripts activated in 8‐cell‐embryo stage. Consistent with KLF3 playing function in 8‐cell embryos, KLF3 protein level is peaked at 8‐cell stage. These data indicate that we have identified a subset of cells that may partially mimic 8‐cell‐embryo stage. Our study identifies a previously unrecognized heterogeneous state of mESCs and highlights the importance of post‐translational regulation in early embryo development.

Previous studies have defined a 2‐cell‐like state based on transcriptional activity of MERVL.^7^ Our study indicates that cells mimicking other stages before blastocyst embryo may exist in mESC cultures. These findings raise an interesting possibility that sequential states mimicking earlier embryo stages prior to blastocyst exist in mESC cultures. Currently, we have no evidence to support that KLF3‐positive cells are direct descendants of 2‐cell‐like state. To test this, time resolved imaging experiments using MERVL and KLF3 reporter cell lines should be performed. In addition, a more pressing question is whether KLF3‐positive cells have any functional importance to the homeostasis of ESC culture. This may be tested by specifically eliminating KLF3‐positive cells from the culture, for example, by fusing KLF3 with a cell killing toxin protein. Interestingly, a previous study shows that *Klf3* homozygous knockout is born at the proportion slightly less than Mendelian ratio,^36^ suggesting a potential function of Klf3 in early mouse embryo development. However, whether this minor defect can be explained by potential function of Klf3 in 8‐cell embryos is not clear. Since KLF family has 17 members,^23^ whether other KLF TFs play redundant functions as Klf3 is still unknown. Answering these questions will likely provide further insights on early mouse embryo development, particularly around 8‐cell‐embryo stages.

Our study reveals that KLF3 protein is translated but significantly degraded in mESCs and C2C12 cells. However, the current data do not exclude other mechanisms inhibiting KLF3 protein expression, for example, translational repression. How KLF3 is degraded warrants further investigations. A potential explanation supported by our data is that the intrinsic disordered structure at the N terminal of KLF3 may cause it inherently sensitive to proteasome degradation.^48^, ^49^ How many other transcription factors are regulated in the same manner as KLF3 is another interesting question. Answering this question also requires the elucidation of exact regulatory pathway that leads to the repression of KLF3. Furthermore, maybe an equally interesting question is how KLF3 is stabilized in cell lineages of skeletal muscle and heart. A ‘nanny protein’^50^ may be co‐expressed with KLF3 in these cells. Therefore, protein pull‐down experiments in these cells will likely provide hints for how KLF3 is stabilized in certain cells. Finally, chromatin immunoprecipitation followed by high‐throughput sequencing needs to be performed for KLF3 in 8‐cell embryos and tissues such as heart and SM. These studies will provide insights not only for the physiologic role of KLF3 in these cells but also for why KLF3 has to be repressed in many other cells.

## CONFLICT OF INTEREST

The authors declare no competing interests.

## AUTHOR CONTRIBUTIONS

JH performed all experiments except these indicated below with help from other authors and bioinformatics analysis. XY initiated the project and performed experiments in Figures 1B,C,2C,7A and RNA‐seq experiments for KLF3 overexpressing mESCs with help from JH. CZ performed experiments in Figure 4B,C. XTZ helped with polysome fraction experiments. MS, LM and YTZ helped with low‐input qRT‐PCR, qRT‐PCR and luciferase experiments. SHW helped with vector construction. YW conceived and supervised the project. YW and JH wrote the manuscript with help from HC, CZ and XY.

## Supporting information

Fig S1‐S7

Table S1

Table S2

Table S3

Supplementary Material

## Data Availability

All data generated or analysed during this study are included in the manuscript and its supplementary information files. RNA‐seq data will be deposited in NCBI’s Gene Expression Omnibus and the accession number will be provided before publication. All data that support the findings of this study are available from the corresponding authors upon request.
