# Supplementary material for: KLF3 promotes the 8‐cell‐like transcriptional state in pluripotent stem cells
Source: Cell Prolif. 2020 Sep 29;53(11):e12914. doi: 10.1111/cpr.12914 (PMC7653263; doi:10.1111/cpr.12914)
Supplement: Supplementary file 1 — Fig S1‐S7 [file CPR-53-e12914-s001.docx]

**Supplementary Figure 1**

**
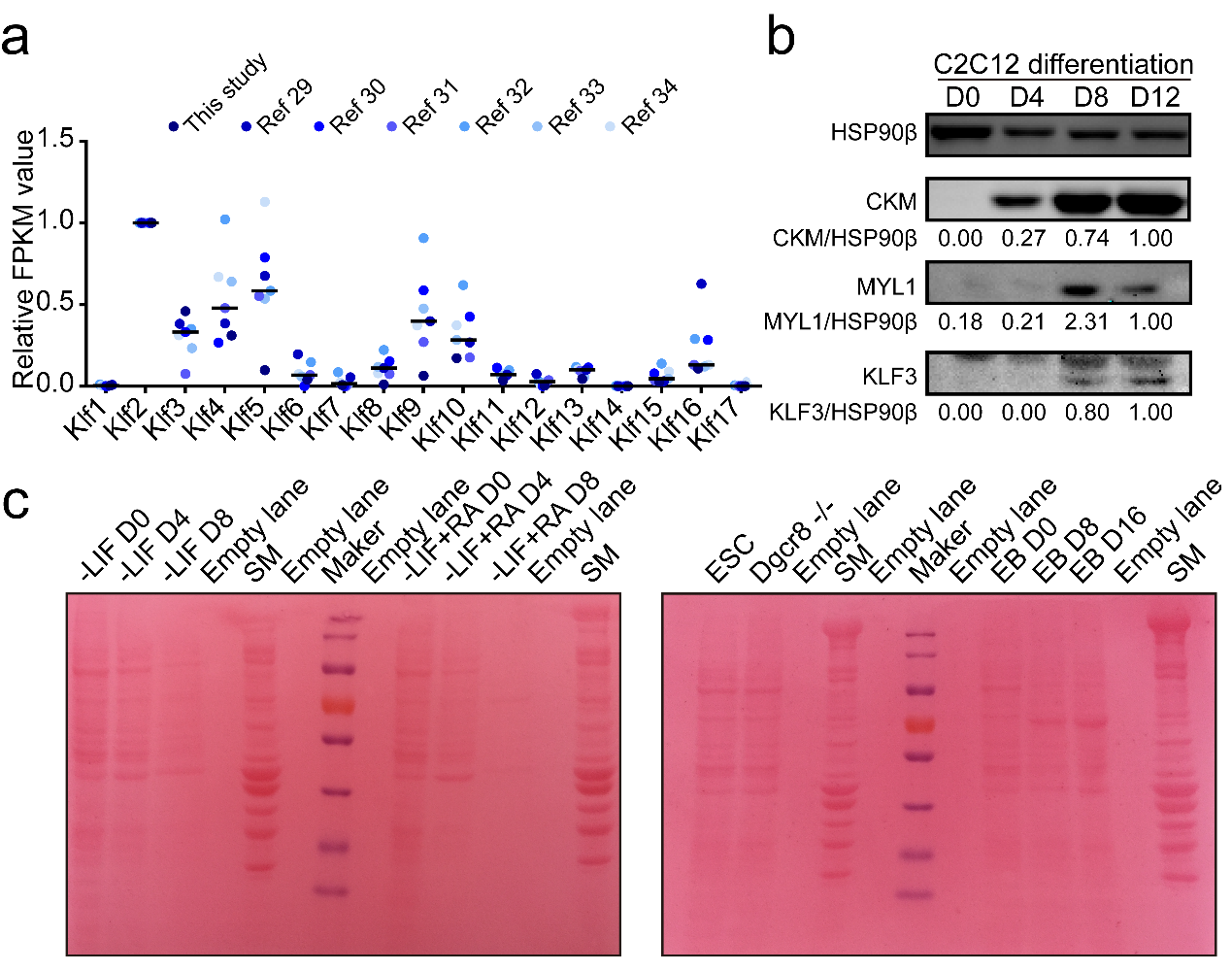
**

**Supplementary Figure 1. The expression of KLF3 protein is repressed in mESCs and C2C12 cells.**

1. Relative RNA expression level of KLF family genes. For each gene, FPKM were normalized to Klf2. Center line, median. References for data source are indicated.
2. Western blotting analysis of KLF3, CKM and MYL1 during C2C12 differentiation. For each sample, 20 μg protein was loaded. Data were quantification of protein level normalized to HSP90β then to day 12 differentiated C2C12.
3. Ponveau S image as input control for Fig. 1e-h.

**Supplementary Figure 2**

**
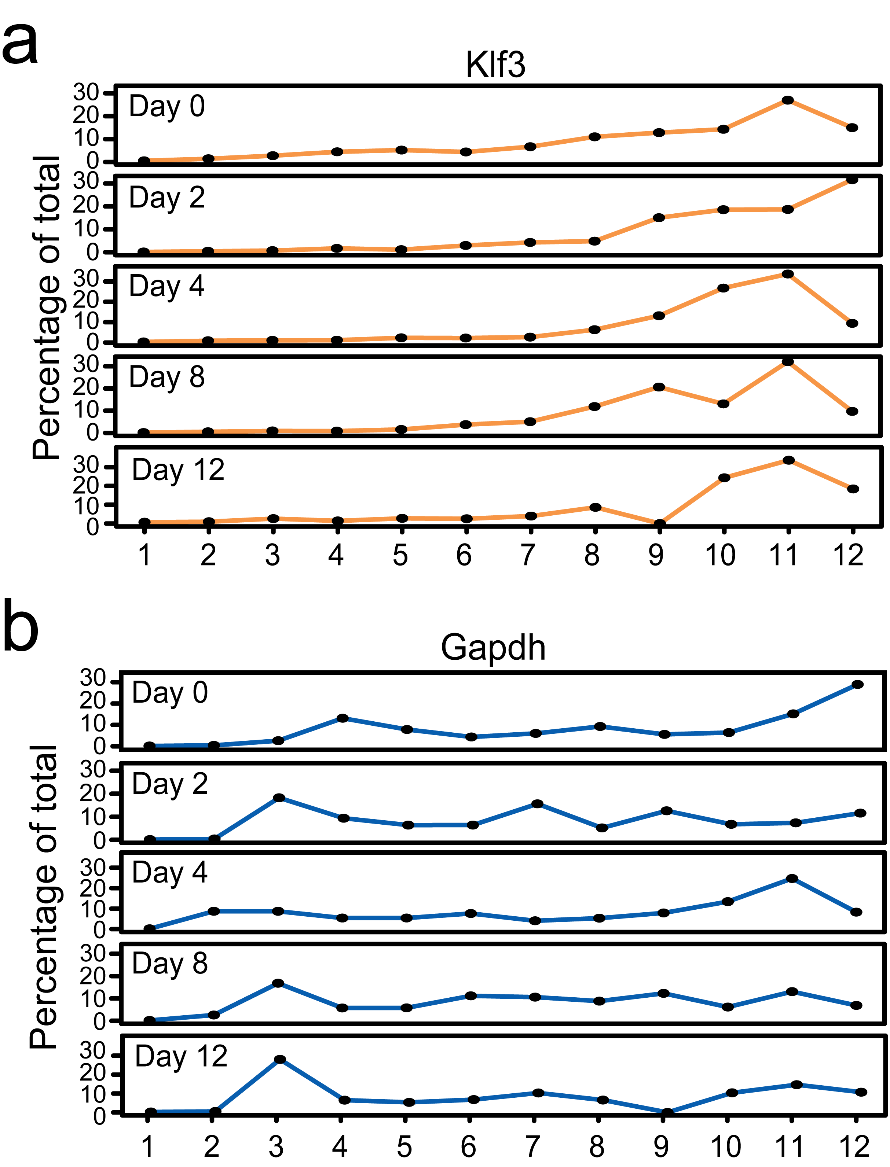
**

**Supplementary Figure 2. Ribosome profiling of Klf3 during C2C12 differentiation.**

**(a-b)** Shown are relative RNA level of Klf3 **(a)** and Gapdh **(b)** in polysome fraction during different time points of differentiation. For each gene, data were normalized to spike-in RNA.

**Supplementary Figure 3**

**
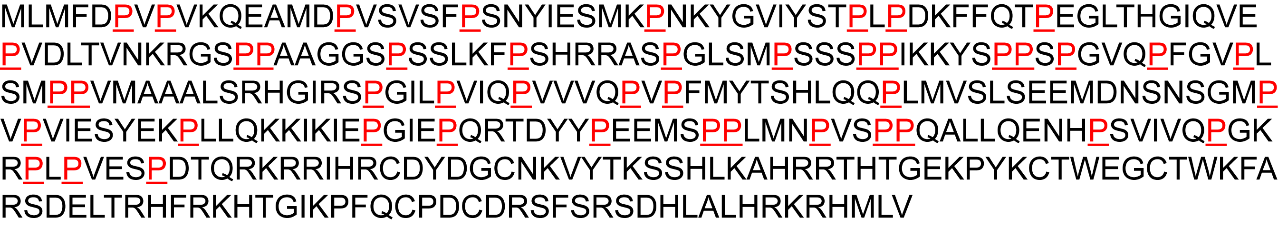
**

**Supplementary Figure 3. Sequences of Klf3 protein.**

Amino acids are shown in single letter code. Prolines are in red.

**Supplementary Figure 4**


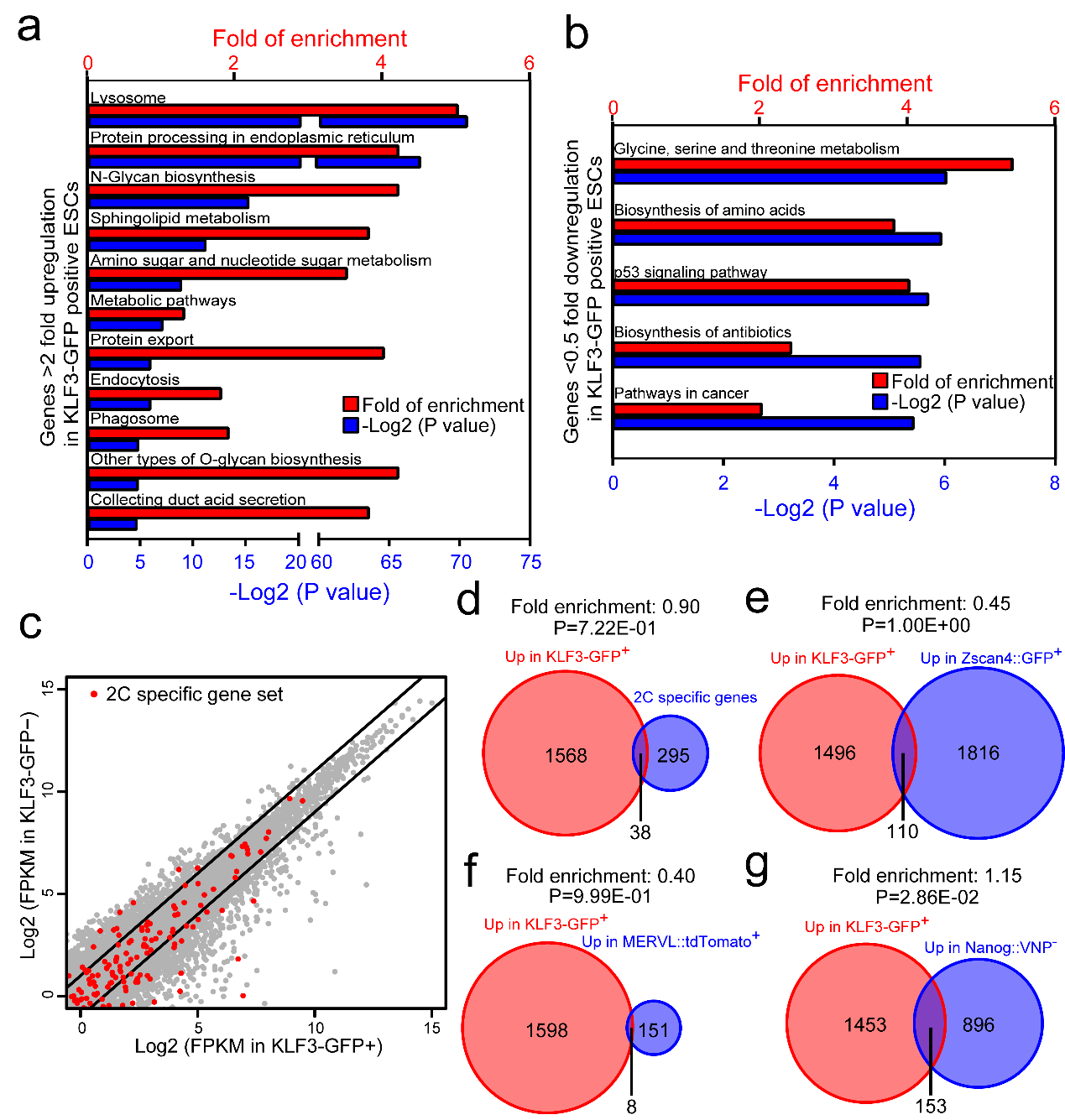


**Supplementary Figure 4. Transcriptome difference between KLF3-GFP positive and negative mESCs.**

1. KEGG pathway analysis of upregulated genes with >2-fold and P value <0.01 in KLF3-GFP positive versus negative mESCs. Shown are P value and fold of enrichment. Enriched pathways with P value <0.05 are listed.
2. KEGG pathway analysis of downregulated genes with <0.5-fold and P value <0.01 in KLF3-GFP positive versus negative mESCs. Shown are P value and fold of enrichment. Enriched pathways with P value <0.05 are listed.
3. Scatter plot of FPKM for genes in KLF3-GFP positive and negative cells. 2C-specific genes are indicated by red dots.

(**d-g**)Venn diagrams showing the overlap between genes upregulated in KLF3-GFP positive mESCs and (**d**) 2C specific genes, (**e**) genes upregulated in Zscan4::GFP positive ESCs, (**f**) genes upregulated in MERVL::tdTomato positive ESCs, or (**g**) genes upregulated in Nanog::VNP negative mESCs. Fold enrichment and P values are shown. The P value was calculated by hypergeometric test.

**Supplementary Figure 5**

**
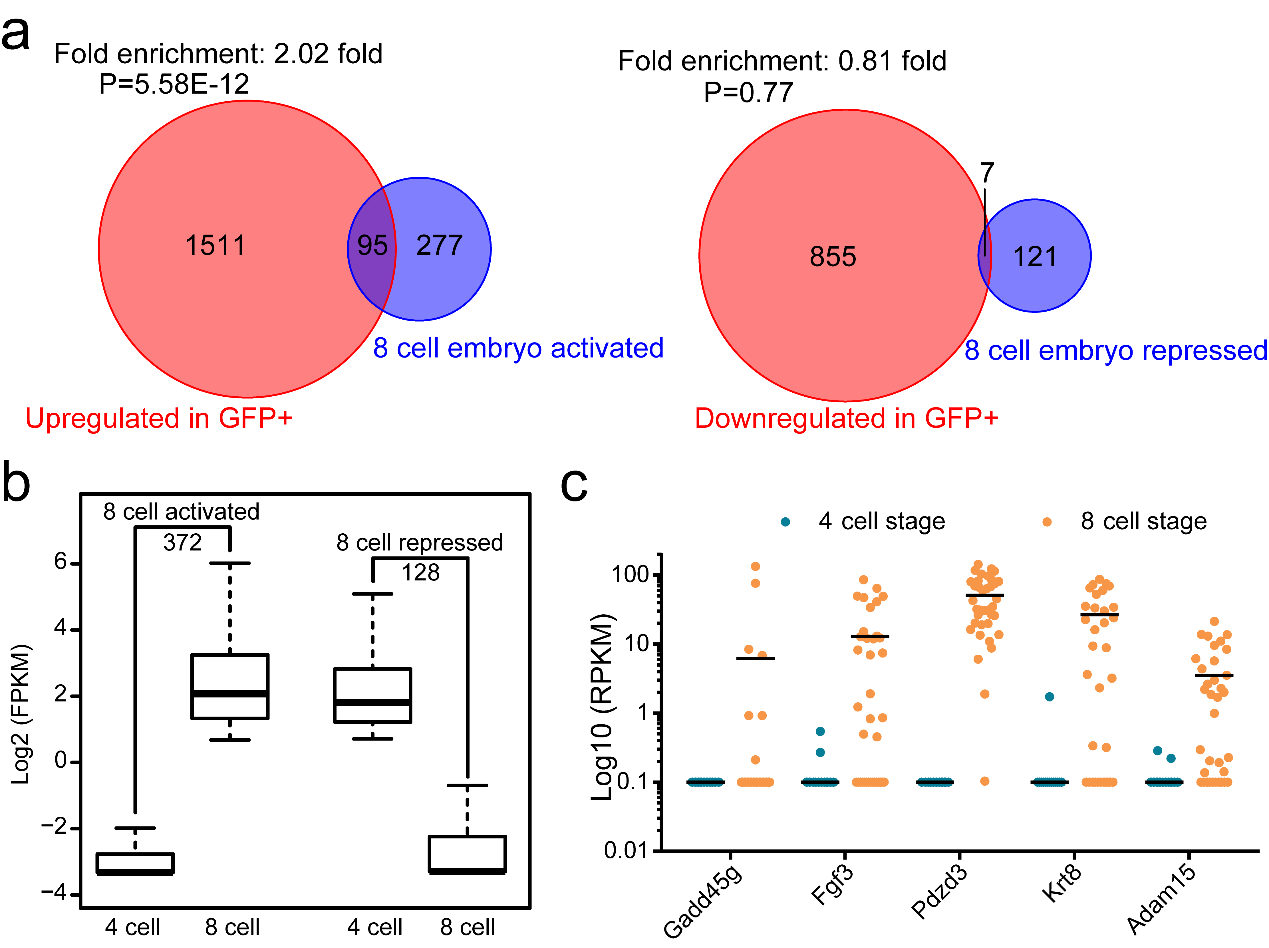
**

**Supplementary Figure 5. Genes activated from 4 cell to 8 cell embryo stage are upregulated in KLF3-GFP positive mESCs.**

1. Venn diagrams showing the overlap between KLF3-GFP positive mESC upregulated genes and 8-cell-embryo activated genes (left) and between KLF3-GFP positive mESC downregulated genes and 8-cell-embryo repressed genes (right).
2. FPKM value for genes activated or repressed at 8 cell stage in 4 or 8 cell embryos. Center line, median; box limits, upper and lower quartiles; whiskers, 1.5× interquartile range.
3. Expression of selected 8-cell-embryo activated genes in single cell RNA-seq. Center line, mean.

For (b) and (c), RNA-seq data are from Ref 41.

**Supplementary Figure 6**

**
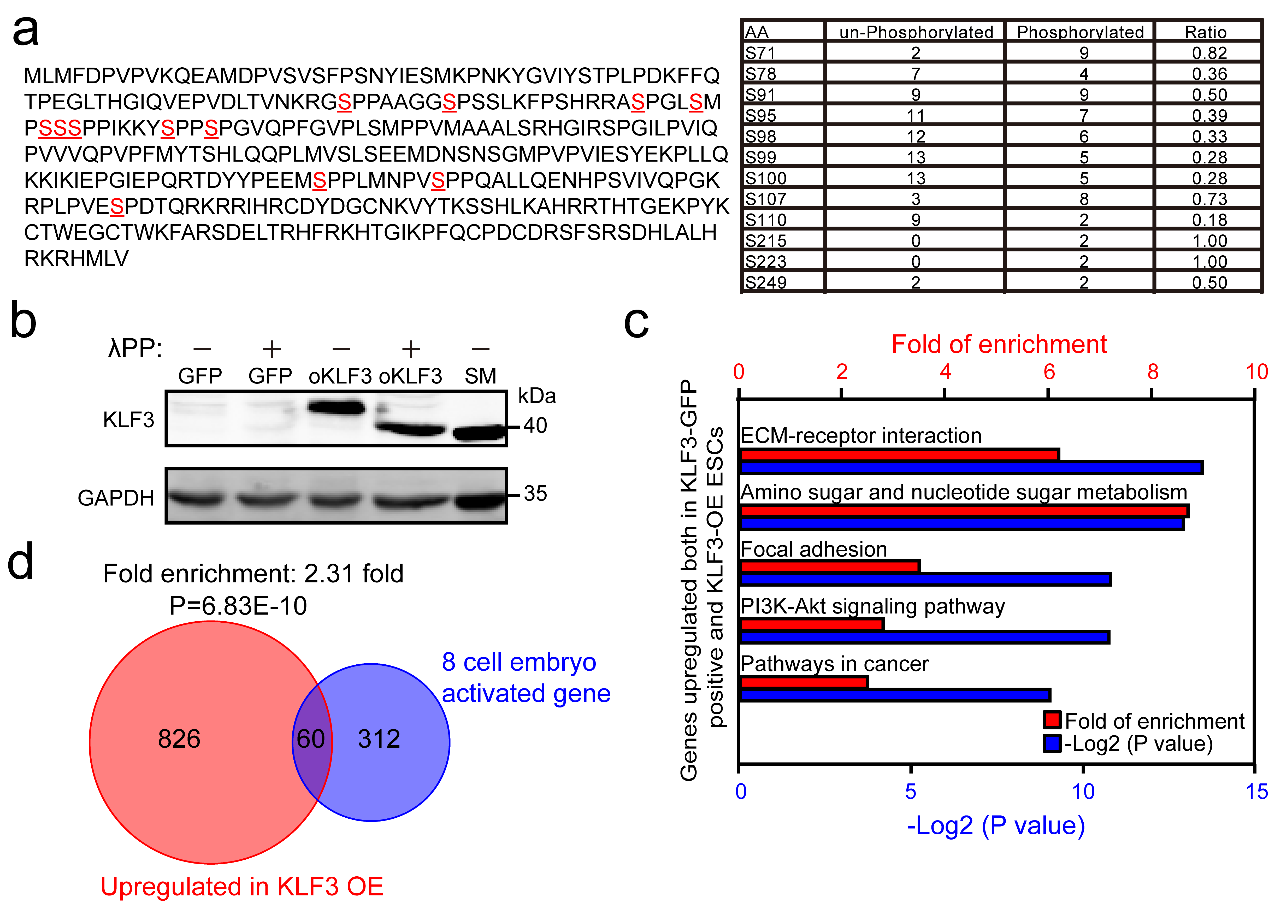
**

**Supplementary Figure 6. KLF3 overexpression upregulates a subset of 8-cell-embryo activated genes.**

1. Mass spectrometry analysis showing that multiple serine of KLF3 were phosphorylated. Left panel shows phosphorylated serine detected by mass spectrum. Right panel shows peptide counts and the phosphorylation ratio of peptides containing indicated serine.
2. Western blotting analysis of KLF3 in GFP or KLF3 overexpressing mESCs treated with λpp. For each sample, 20 μg protein extract was loaded except SM, for which 5 μg protein extract was loaded.
3. KEGG pathway analysis of genes upregulated in both KLF3-GFP positive and KLF3 OE mESCs. Shown are P value and fold of enrichment. Enriched pathways with P value <0.05 are listed.
4. The Venn diagram shows the overlap between 8 cell-embryo-activated genes and genes upregulated in KLF3 overexpressing mESCs. Fold enrichment and P value are shown. The P value was calculated by hypergeometric test.

**Supplementary Figure 7.** Uncropped western blotting images for main and supplementary figures.

**
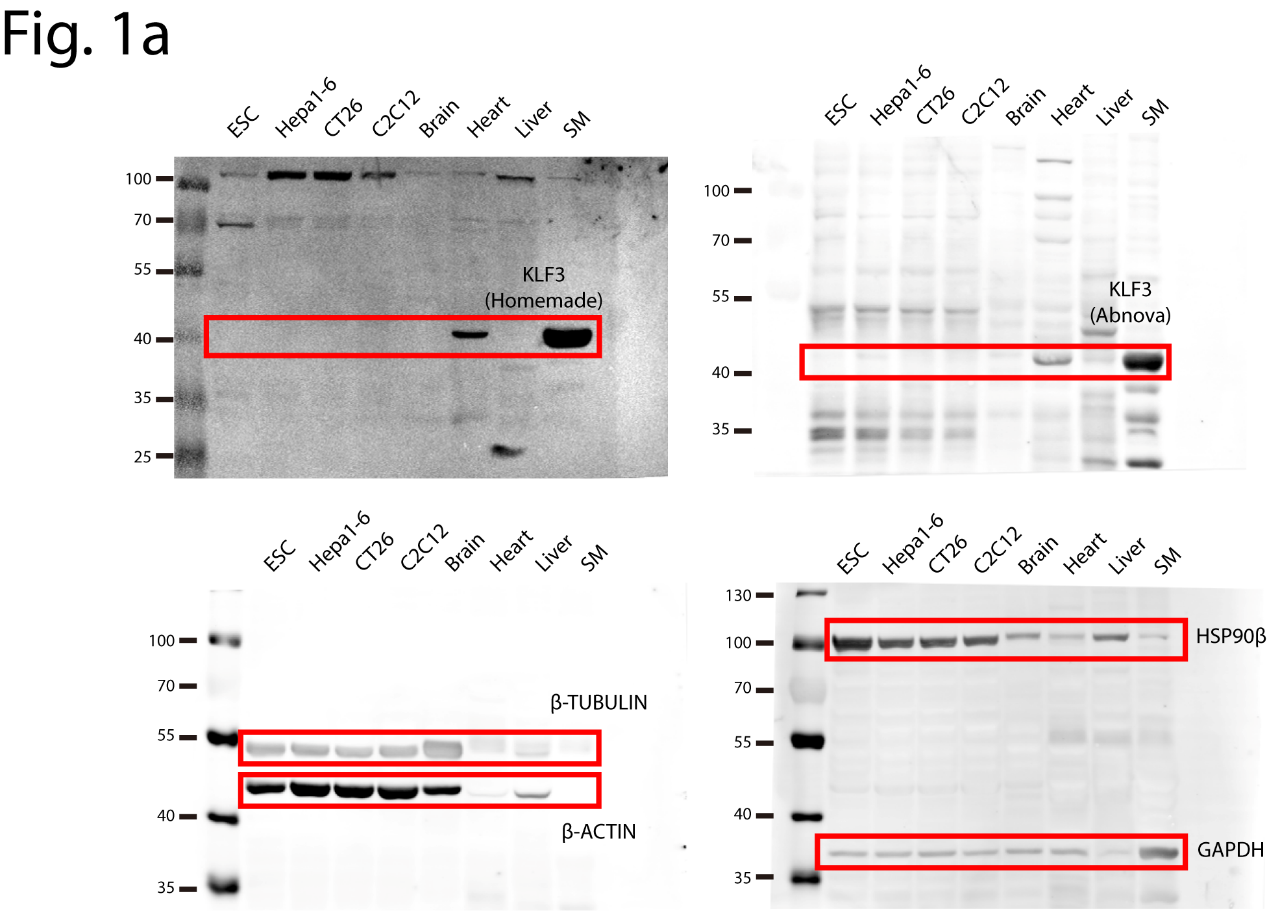
**

**
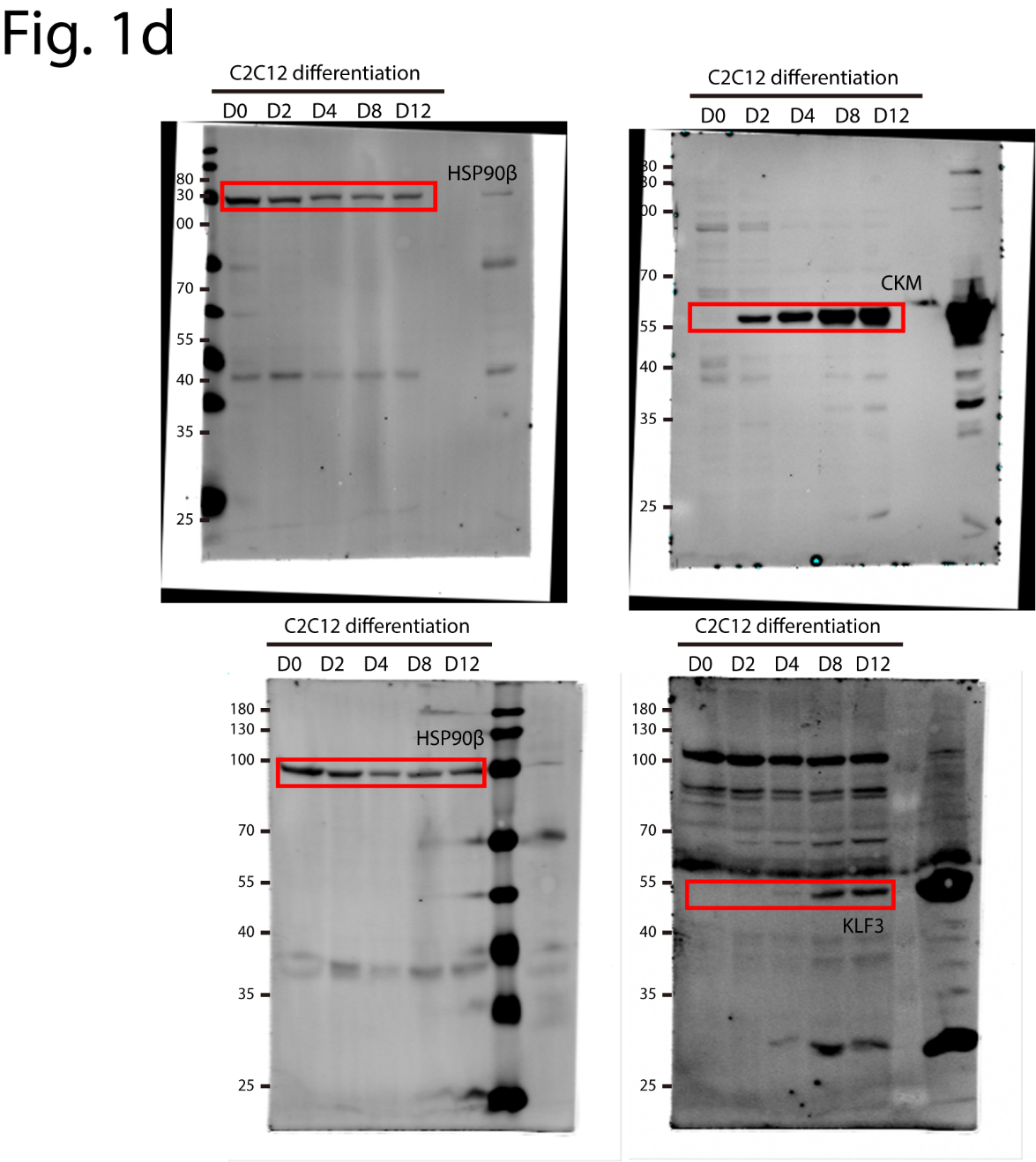
**

**
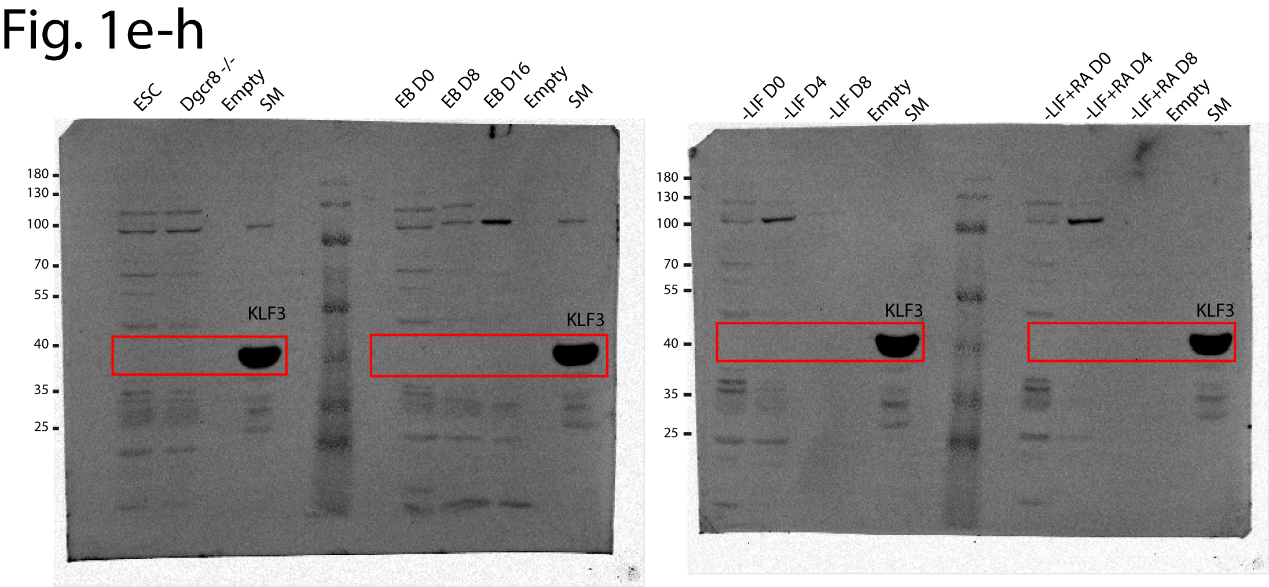
**

**
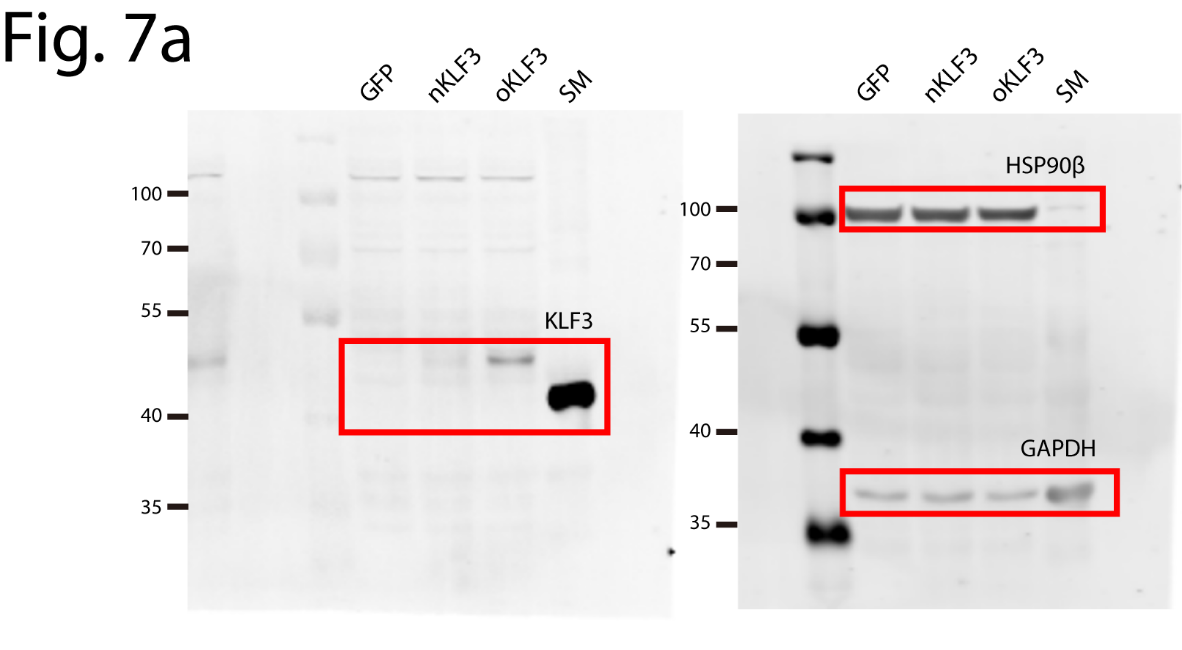
**

**
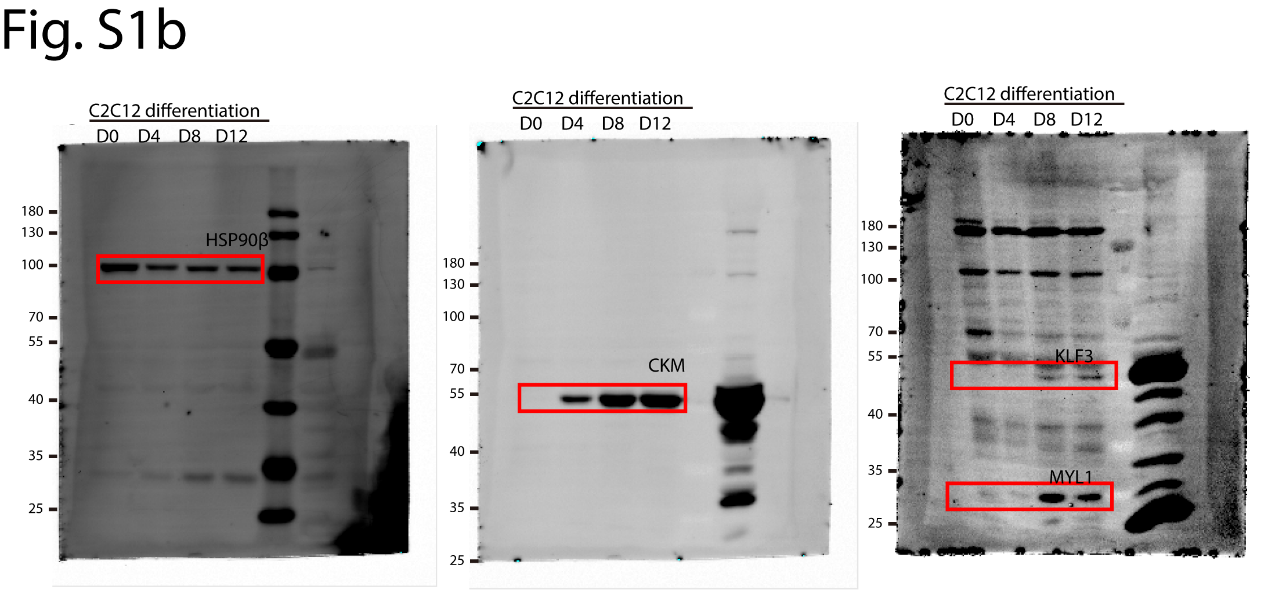
**

**
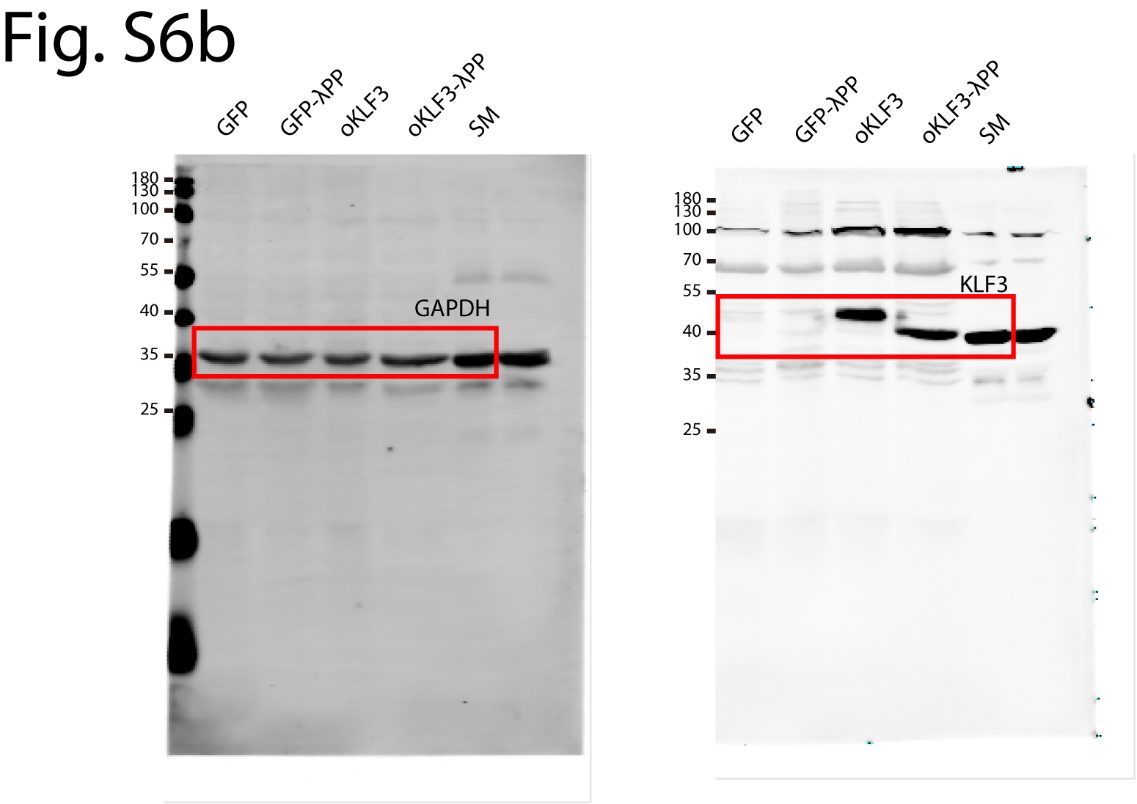
**
